# The Impact of Low-Lactose, High Galacto-Oligosaccharides Milk on Gut Microbiome and Plasma Metabolome in Healthy Adults: A Randomized, Double-Blind, Controlled Clinical Trial Complemented by Ex Vivo Experiments

**DOI:** 10.1016/j.cdnut.2025.107506

**Published:** 2025-07-24

**Authors:** Léa Siegwald, Anna Cherta-Murillo, Stefan Christen, Claire L Boulangé, Chieh J Chou, Francis Foata, Anirban Lahiry, Adrien Frézal, Maria Pilar Giner, Jean-Philippe Godin, Olga Sakwinska

**Affiliations:** 1 Nestlé Institute of Health Sciences, Nestlé Research, Lausanne, Switzerland; 2 Nestlé Institute of Food Safety and Analytical Sciences, Nestlé Research, Lausanne, Switzerland; 3 Clinical Research Unit, Nestlé Research, Lausanne, Switzerland

**Keywords:** galacto-oligosaccharides, milk, short-chain fatty acids, microbiome, Bifidobacterium, lactose sensitive, low-lactose, 3-indole propionate, tryptophan metabolism

## Abstract

**Background:**

Galacto-oligosaccharides (GOS) intake has been linked to health benefits via modulation of the gut microbiome. Milk, where the majority of lactose is enzymatically converted to GOS (called here Novel or “N milk”), retains milk’s nutritional value with reduced lactose and a high amount of prebiotic GOS.

**Objectives:**

The aim of this study was to investigate the effect of N milk on the gut microbiome and related changes in health-related biomarkers, complemented by ex vivo fermentation experiments.

**Methods:**

In a 2-arm crossover, double-blind, randomized controlled clinical trial, 26 healthy adults consumed either N milk (containing 9 g GOS and 1.7 g of lactose per serving) or lactose-free milk (control), for 2 wk with a 2-wk washout period. Stool and fasting blood samples were collected at the start and the end of the intervention periods. Gut microbiome was analyzed using shotgun metagenomics, and metabolites using both targeted and untargeted methods. In addition, we tested lactose-free milk, N milk, and GOS in ex vivo colonic fermentation to obtain insights into the bacterial processing of substrates.

**Results:**

N milk intake led to a 3-fold increase in median gut bifidobacteria (*P* < 0.0001) and significant increases in plasma acetate, octanoic acid, β-alanine, and nicotinamide (all *P* < 0.05). Untargeted plasma metabolomics revealed a shift in amino acid metabolism, with an increase in 3-indole propionate, accompanied by a decrease in 2 uremic toxins, p-cresol sulfate, and indoxyl-sulfate (*P* < 0.05 without false discovery rate adjustment). Ex vivo fermentation experiments supported the results of the clinical study, whereby N milk increased bifidobacteria accompanied by higher production of short-chain fatty acids and a shift in microbial tryptophan metabolism, and indicated unique effects of N milk compared with GOS.

**Conclusions:**

N milk resulted in a significant increase in gut bifidobacteria, along with changes in plasma metabolites previously associated with immune and metabolic health benefits.

This study was registered at clinicaltrials.gov as NCT05207839.

## Introduction

Dietary fiber is a fundamental component of a healthy and balanced diet, and as such, its recommended intake is stipulated by international and national guidelines [1]. Many governmental and international bodies recommend daily intakes between 21 and 38 g of fiber for adults (WHO: 25 g; United States: 21–38 g; United Kingdom: 30 g; and China: 25–30 g [2]). However, daily fiber intakes generally fall short of these targets [[3], [4], [5]]. The barriers to an adequate intake include the intrinsic characteristics of fiber itself, such as taste, texture, and consumer appeal [6]. Furthermore, consumption of prebiotic fiber may lead to gut discomfort (e.g., bloating, cramping, and sensation of fullness) and flatulence, particularly with certain types of fiber, which may further contribute to intakes below the recommended levels [7].

One of the key mechanisms of action linking fiber consumption with health outcomes is the production of health-linked metabolites by the gut microbiome. There is accumulating evidence on the role of the gastrointestinal microbiome in metabolic, immunologic, and endocrine functions of the host [[8], [9], [10], [11], [12]]. Indeed, the fermentation of fiber by the gut microbiome produces short-chain fatty acids (SCFA), including acetate, lactate, propionate, and butyrate [13,14], which are associated with various health benefits [1]. SCFA trigger signaling in metabolically active tissues via G-protein-coupled receptors, such as in the liver, skeletal muscle [15], adipocytes, pancreatic cells [16], and the hypothalamus in the brain [17], as well as via GPCR-independent mechanisms in immune cells [18], thereby playing a pivotal role in health. Other microbially derived metabolites, such as indole derivatives, have also been shown to play a role in the physiology of the host [[19], [20], [21], [22], [23]].

β-Galacto-oligosaccharide (β-GOS), herein called GOS, is a well-studied prebiotic fiber derived from lactose [24]. Previous studies have shown that GOS supplementation led to an increase in gut bifidobacteria, sometimes linked to a change in metabolic health markers [[25], [26], [27], [28]]. Bifidobacteria are efficient utilizers of GOS, capable of fermenting a wider diversity of structures than other gut microbes [29]. GOS is produced by several types of bacteria naturally present in milk and fermented milk products, but only in minor amounts (e.g., 60–350 mg/L in mature goat milk [30] and 0.33–0.71 g/100 g in fermented products such as yogurts [31]).

Milk consumption is associated with a lower risk of cardiovascular disease, stroke, hypertension, metabolic syndrome, obesity, and osteoporosis [32]. The health benefits linked to milk intake are linked to its nutritional value. Milk proteins are of high quality and biological value, in addition to containing several bioactive peptides (such as α-lactalbumin and β-lactoglobulin) [33]. In addition, milk is a good source of calcium, phosphorus, magnesium, zinc, and selenium as well as vitamins A, D, E, and B-complex vitamins such as thiamin and riboflavin [32]. Despite its high nutritional value, milk consumption in adults has declined in recent decades [34]. One of the potential underlying reasons may be linked to concerns regarding lactose intolerance, although compelling evidence shows that small to moderate amounts of lactose can be consumed by lactase intolerant individuals without any symptoms [35]. Cultural trends, concerns about carbon footprint, and lack of awareness of the high nutritional value of milk may also play a role in the declining milk and dairy consumption, although dietary guidelines of nutrition societies worldwide recommend higher dairy consumption than current intakes [2].

Providing milk with a substantial dose of prebiotic GOS fiber could bring substantial nutritional benefits, leading to health improvements over the longer term. We hypothesized that short-term intake would lead to a robust increase in gut bifidobacteria and concomitant improvement in microbially produced compounds, previously linked to health benefits. To this end, we tested a novel milk formulation where most lactose was converted to GOS via 1-step in situ trans-galactosylation. The resulting milk (herein called Novel or “N milk”) features a specific carbohydrate profile low in lactose and high in GOS while preserving other milk nutrients. In addition, N milk contains a lower amount of monosaccharides in comparison to a typical composition of lactose-free (LF) milk, where lactose is hydrolyzed to galactose and glucose. We hypothesized that the effect of N milk would reflect known benefits of GOS, with a potential for a synergistic effect with favorable carbohydrate composition and other beneficial milk components.

In the clinical study, we compared the effects of a dietary intervention with 1.5 servings of N milk to LF milk, focusing on the impact on gut microbiome, and exploring plasma metabolome in healthy adults. The clinical study was complemented by an ex vivo fermentation approach to gain insights into how N milk modified the composition and functional capacity of the colonic microbiome. Including both GOS (not embedded in the milk matrix) alongside N milk and LF milk as control allowed the detection of potential synergistic effects between GOS and other milk components.

## Methods

### Clinical study

#### Study design and participants

This study was a monocentric, double-blind, crossover, randomized controlled clinical trial conducted at the Clinical Innovation Lab at Nestle Research (Lausanne, Switzerland) in October–November 2022. The study was conducted according to the guidelines from the Declaration of Helsinki and Good Clinical Practice. All procedures involving human subjects were approved by the Canton of Vaud Research Ethics Committee (num. ID 2021-01458). The protocol of the trial was registered at www.clinicaltrial.gov (NCT05207839). A total of 33 volunteers were recruited and screened for the study. Participants were screened against the eligibility criteria described in Supplemental Methods and randomly assigned to the investigational product or control via dynamic allocation randomization performed using the software Medidata Rave RTSM. Participants were randomly allocated to 1 of the 2 possible sequences. Among the 24 included in the statistical analyses, 12 were allocated to the “N milk → Control” sequence and 12 were allocated to the “Control → N milk” sequence. Allocations were blinded for both participants and researchers. Both interventions were identified with a unique code linked to the allocation and trial participant ID. The treatment code was not broken as there were no medical emergencies or a need to report unexpected and related severe adverse events to the Research Ethics Committee. Further details on study procedures are provided in Supplemental Methods.

#### Study procedures

At screening (visit 1), the protocol was explained in full, and signed informed consent forms were obtained. Next, participants underwent demographic, medical history, and inclusion/exclusion criteria questions, followed by a urine pregnancy test for women of reproductive age. Eligible participants were invited for 5 more study visits. At initiation (visit 2), participants were randomly assigned, and a stool and saliva sample collection kit was provided with instructions on collection procedures. A food frequency questionnaire was also completed during this second visit. Ahead of baseline study visit 3, the participants collected stool and saliva samples at home. Fasting blood samples were obtained at visit 3 after an overnight fast. Participants completed the questionnaires (via Medidata ePRO application), including Gastrointestinal Symptom Rating Scale (GSRS), State-Trait Anxiety Inventory, bowel movement pattern, stool consistency (based on Bristol stool scale [[36], [37], [38]]), and Short Form Health Survey during baseline study visit 3. After study visit 3, participants were instructed to consume the products once a day. On day 14, participants attended visit 4, which was of the same nature as visit 3, followed by a 14-d washout period, before the next 14-d intervention period commenced, with visits 5 and 6 at the start and end of the period, respectively. Visits 5 and 6 were of the same nature as visits 3 and 4. Product dispensation for 14 d occurred at visits 3 and 5. Adverse events, concomitant medication, and compliance of the product throughout the study were assessed at each study visit. Supplemental Figure 1 illustrates the study design.

#### Sample size

This study was exploratory; hence, no formal power calculation was performed. We based our sample size on similar crossover studies observing a significant increase in the proportion of *Bifidobacterium* after the intake of fiber-rich products [39], leading to 20 subjects to be recruited. On the basis of previous experience of similar trials, a 30% drop-out was expected, leading to the enrollment of 26 participants.

#### Investigational products

The investigational product was N milk powder. N milk powder was generated in-house in a 1-step in situ trans-galactosylation, where ∼80% of lactose was converted to a blend of GOS with small amounts of glucose and galactose (Table 1). The control product was LF skim milk powder. Both products were given in a sachet containing 33 g of milk powder, which the participants were instructed to reconstitute in 200 mL of water. Both products were isovolumetric, energy-, fat-, and protein-matched and had similar taste and smell.

**TABLE 1:** Nutritional composition of investigational and control products.

	Per 100 g dry weight		Per serving size (33 g)	
	Control (lactose-free skim milk powder)	Investigational (N milk powder)	Control (lactose-free skim milk powder)	Investigational (N milk powder)
Energy (kcal)	351	364	115	120.1
Carbohydrate (g)	51.1	54	16.9	17.8
Of which sugars (g)	51.1	20	15	6.6
Of which lactose (g)	0	6	<0.1g	2
Fat (g)	0.29	1	<0.5	<0.5
Protein (g)	34.1	34	10.2	11.2
Galacto-oligosaccharides (g)	0	30	0	9.9

#### Analysis of stool samples

DNA was extracted from fecal samples using the QIAamp DNA Stool minikit (Qiagen) with the addition of mechanical disruption steps (4 × 60 on FastPrep with Lysing Matrix B tubes from MP Biochemicals). DNA concentration was adjusted to 10 ng/μL for sequencing library preparation. High-throughput sequencing was performed on an Illumina NextSeq2000 with a PE150 approach, pooling data from 2 sequencing runs to achieve an output of around 10 Gb per sample. Adaptor sequences were trimmed from raw sequencing reads, and host sequences mapping the human genome were removed. Taxonomic profiles at the species level were generated using MetaPhlAn v4.0.6 [40] using the mpa_vOct22_CHOCOPhlAnSGB_202212 database, and MetaCyc pathway profiles were generated using HUMAnN v3.7 [41].

Each stool sample was homogenized and divided into 2 subsamples. Fecal SCFA were extracted from the first subsample using a solution of 0.1% ortho-phosphoric acid to prepare fecal water, whereas the water content of each sample was determined from the second subsample through a freeze-drying process. The quantification of SCFA was performed by GC-MS after derivatization of the compounds in the fecal water using an automated platform. The method is detailed in the Supplemental Methods.

#### Analysis of plasma and serum samples

Plasma SCFA analysis was performed by liquid chromatography tandem mass spectrometry (LC-MS/MS) according to Valdivia-Garcia et al. [42]. Derivatized compounds were detected and quantified in negative mode using multiple reaction monitoring transitions. The analytes were normalized and quantified using calibration curves constructed with isotopically labeled internal standards.

Plasma water-soluble vitamins were measured by LC-MS/MS. Sample preparation, including calibration curves, quality controls, and study samples, was automatically performed on a Microlab Star M liquid handler (Hamilton) with ascorbic acid, DL-dithiothreitol, and internal standards as additives. The analyses were performed on an Acquity*I*-class ultra-high-pressure liquid chromatography (UPLC) system (Waters) hyphenated to an Xevo TQ-XS triple quadrupole mass spectrometer (Waters). Separations were performed on an ACE Excel C_18_-PFP column (100 × 2.1 mm, 2 μm, ACE) with a gradient using 5% acetic acid with 0.2% heptafluorobutyric acid in Milli-Q water, and acetonitrile as mobile phases. Data were acquired using MassLynx software (Waters), and chromatographic peaks were integrated with TargetLynx (Waters).

Serum amino acid processing, including a derivatization step, was automated and carried out on a Microlab Star M liquid handler (Hamilton). The protein of the plasma samples was precipitated, and the supernatant was derivatized in borate buffer (pH 8.8) with aminoquinolyl-*N*-hydroxysuccinimidyl carbamate at 55°C for 10 min. Finally, samples were diluted 50 times with a 10 mM ammonium formate and 0.1% formic acid before LC-MS/MS analysis. Amino acid analysis was performed on an Acquity *I*-class UPLC system (Waters) hyphenated to a Xevo TQ-XS triple quadrupole mass spectrometer (Waters). Separation was performed on an AccQtag Ultra C_18_ column (2.1 × 100 mm, 1.7 μm, Waters). Data were analyzed with MassLynx software (Waters), and chromatographic peaks were integrated with TargetLynx (Waters).

Additional serum analytes were directly measured without prior preparation, including holotranscobalamin, folate, homocysteine, magnesium, and copper. Clinical chemistry analyses were carried out on an Architect Ci4100 analyzer (Abbott) composed of a C4000 clinical chemistry module and an i1000SR immunoassay module. Samples exceeding the upper limit of quantification were diluted by a factor of 2 according to the recommended instructions and reanalyzed. The full list of analytes measured with targeted methods is listed in Supplemental Table 1.

#### Untargeted plasma metabolomics

A total of 15 μL plasma was extracted with 500 μL cold –20°C acetonitrile/methanol/H_2_O (40/40/20, v/v/v) containing isotopically labeled standards. Pooled plasma served as quality control, where different amounts (5, 10, 15, 20, and 25 μL) were processed as described above. After drying, samples were resuspended in acetonitrile/H_2_O (70/30, v/v) before analysis by LC-MS. The chromatographic system (Vanquish UPLC, Thermo Scientific) was connected to an Orbitrap mass spectrometer (Orbitrap Fusion Lumos Tribrid, Thermo Scientific) equipped with a heated electrospray ionization source operating in negative and positive ionization modes. Separation was achieved with a hydrophilic interaction chromatography analytical column (2.1 mm × 100 mm, 5 μm pore size, 200 Å, ZIC-pHILIC). Aacetonitrile and H_2_O containing 10% ammonium acetate and 0.4% ammonium hydroxide served as elution buffers. The data were processed with an automated pipeline in R (version 3.6.2) (Supplemental Methods). The untargeted plasma dataset was log2-transformed to achieve a normal distribution for further analysis.

### *Statistical analysis*

The primary outcome was the change in total bifidobacteria proportion in stools between the 2 groups. Secondary outcomes were quality of life, plasma and stool SCFA concentrations, and GSRS. Linear mixed effects models were implemented to analyze the difference in change from baseline between control and N milk groups for the aforementioned outcomes. Group, sequence, and period were included as fixed effects, and participants nested within sequence as a random effect. Lack of impact of the sequence effect confirmed the absence of carryover between the 2 phases of the trial. Similarly, untargeted plasma metabolomics data were analyzed with linear mixed effects models, including group, sequence, and period as fixed effects and participants nested within sequence as a random effect. Associations between the microbiome at the genus level and plasma SCFA and ketone body concentrations were examined using a Pearson correlation.

### Ex vivo fermentation experiments

An ex vivo batch culture fermentation experiment was performed to gain mechanistic insights into the effects of N milk. Details of the experimental system are described in Supplemental Methods, and Supplemental Figure 2 depicts the study design. Briefly, the conditions tested included control (no substrate control, NSC), LF milk, GOS (GOS-950P), and N milk (Table 1). Samples from 12 adult healthy donors, 25–65 y old, with a BMI <30 kg/m^2^, having no gastrointestinal disorders and receiving no antibiotics 3 mo before participation were used. Fecal samples were collected according to a procedure approved by the Ethics Committee of the University Hospital Ghent (reference number BC-09977). Samples were digested according to the INFOGEST 2.0 method (44; and details in Supplemental Methods). Colonic fermentations were performed with Systemic Intestinal Fermentation Research [43]. The dose of GOS alone and GOS within N milk was 4.3 g/L in fermenters. No residual lactose was detected in N milk after the digestion step. Media samples were collected at 0 h for No Substrate Control (NSC) and at 24 h of fermentation for all conditions. SCFA were quantified using GC-MS [44] at 0 h for NSC, whereas microbiome, metabolomic analyses, and SCFA quantification were performed at 24 h for all conditions.

### Microbiome analysis

DNA was extracted from a bacterial cell pellet (centrifugation of 1 mL sample for 5′ at 9000 × *g*) via the SPINeasy DNA Kit for Soil (MP Biomedicals), according to manufacturer’s instructions. Subsequently, sequencing was performed on an Illumina MiSeq platform PE300 with v3 chemistry. The 16S rDNA gene V3-V4 hypervariable regions were amplified using primers 341F (50 -CCT ACG GGN GGC WGC AG-30) and 785Rmod (50 -GAC TAC HVG GGT ATC TAA KCC-30). Sequencing results were analyzed using the implementation of DADA2 [45] in qiime2 v.2020.11 [46]. Taxonomy was assigned to Amplicon Sequence Variants (ASVs) using the classify-sklearn naïve Bayes taxonomy classifier [47] against the Greengenes 13_8 99% Operational Taxonomic Units (OTUs) reference sequences [48]. Most taxonomic assignments are reported at the genus level, in line with the resolution typically achievable with 16S rDNA V3–V4 sequencing. However, to enable more refined identification of *Bifidobacterium* ASVs, we applied a targeted annotation approach based on the method described by Kieser et al. [49], which allows species- and subspecies-level classification within this genus.

### Semitargeted metabolomics analysis of fermentation samples

Semitargeted metabolomics of the fermentation supernatants by LC-MS analysis was carried out using Vanquish liquid chromatography (Thermo Scientific) coupled to Q Exactive HF MS (Thermo Scientific) operating in negative and positive ionization modes. The UPLC method was adopted from Doneanu et al. [50]. Peak areas were extracted using Compound Discoverer 3.1 (Thermo Scientific). In addition to the automatic compound extraction by Compound Discoverer 3.1, a manual extraction of compounds included in an in-house library was performed using Skyline 21.1 (MacCoss Lab Software, University of Washington) [51]. Metabolomics data were mean-centered and log-transformed before further analysis.

### *Statistical analysis*

For the statistical evaluation of the treatment effects on key fermentation parameters and microbiome composition, a repeated measures analysis of variance was performed. The statistical significance of the potential treatment effects was determined via Benjamini–Hochberg post hoc testing. Comparisons of treatment groups at 24 h for key fermentation parameters, metabolites, and microbiome composition at genus level (clr-transformed) were performed using a paired t-test with false discovery rate (FDR) correction.

## Results

### Clinical study

#### Study population

A total of 33 volunteers were screened, of which 26 healthy adults were enrolled and 24 were randomly assigned to receive the investigational and control products. The CONSORT [52] diagram describes the flow of participants (Supplemental Figure 3). All randomly assigned participants completed the study, and there were no dropouts. The demographic and dietary intake baseline characteristics of the participants are described in Supplemental Tables 2 and 3. Of note, at baseline, the study population had a fiber intake below recommendations, with an average of 12.06 g a day.

#### The effect on microbiome composition and functionality

**FIGURE 1:**
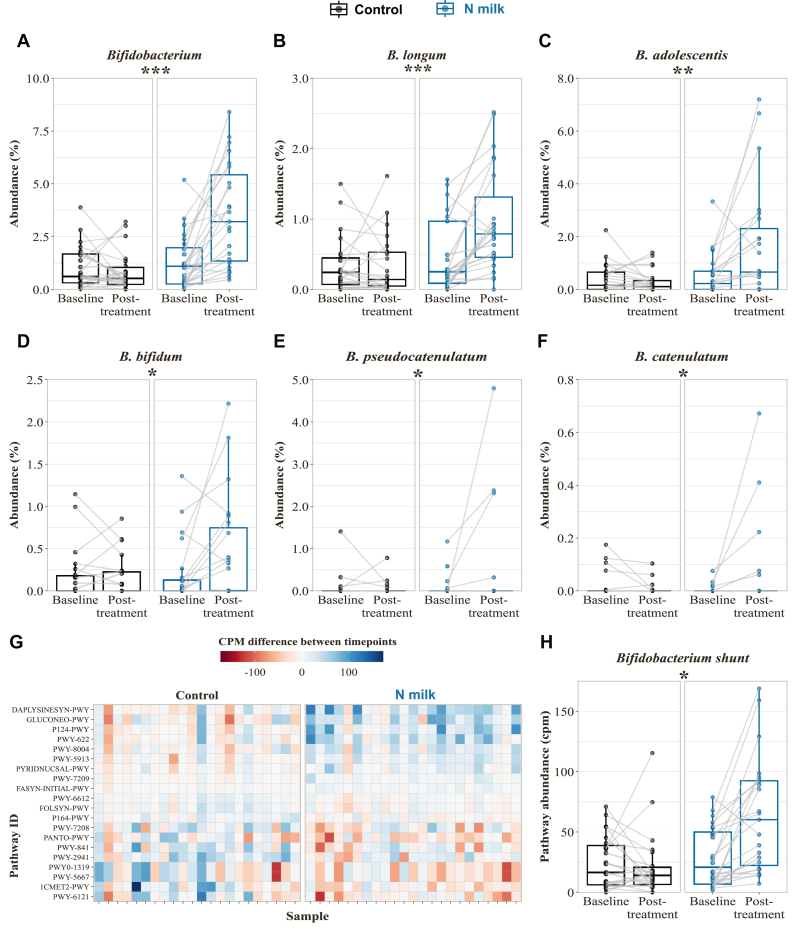
Impact of the intervention on bifidobacteria and microbiome functionality. (A) Bifidobacterium genus and (B–F) Bifidobacterium species for which the abundance difference between before and after timepoints is significantly changed between treatment groups. Phases are merged for clarity (baseline = V3 and V5, posttreatment = V4 and V6). (G) Functional pathways significantly different between control and intervention. (H) Focus on the Bifidobacterium shunt pathway P124-PWY. Statistical differences between groups are visualized via ∗ (0.01 < FDR-adjusted *P* < 0.05), ∗∗ (0.001 < FDR-adjusted *P* < 0.01), or ∗∗∗ (FDR-adjusted *P* < 0.001). Exact statistical results are reported in Supplemental Table 5. FDR, false discovery rate.

We characterized the gut microbiome composition and functional capacity through shotgun metagenomics, focusing on bifidobacteria taxa changes as a primary outcome (Supplemental Table 4). Our study revealed a significant increase in the *Bifidobacterium* genus with N milk compared with control [effect size of differences (clr-transformed %) = 2.62, 95% confidence interval: 1.95, 3.29, *P* = 8.6 × 10^–8^, Figure 1A], with a return to baseline levels after the washout period (no carryover effect was observed). This increase was observed for all *Bifidobacterium* species that were most prevalent in the set of subjects (*Bifidobacterium longum, B. adolescentis, B. bifidum, B. pseudocatenulatum,* and *B. catenulatum*) with effect sizes of differences (clr-transformed %) ranging from 0.98% to 2.02% and FDR-adjusted *P* <0.07 (Figure 1B–F, Supplemental Table 5). When running the statistical model on all taxa, of 734 other detected genera besides *Bifidobacterium*, only *Mediterraneibacter* displayed a significant difference between the 2 groups (FDR-adjusted *P* = 0.028), but the difference was not significant at the species level (Supplemental Table 5). At the species level, besides the *Bifidobacterium* species depicted in Figure 1B–F, only *Clostridium_sp_AF20_17LB* and *Ruminococcus torques* showed significant differences between the control and treatment group (FDR-adjusted *P* = 0.067, Supplemental Table 5). There were no significant differences in overall richness changes (observed species, Chao1, *P* > 0.7) nor diversity changes (Shannon, Inverse Simpson, *P* > 0.4) between the 2 treatment groups (Supplemental Table 4).

Looking at the functional potential of the microbiome, 20 bacterial pathways were significantly differently affected between control and intervention (Figure 1G, Supplemental Table 5), most of them related to carbohydrate metabolism. One of the most increased functional capabilities in the intervention (N milk) group is the *Bifidobacterium* shunt pathway P124-PWY (FDR-adjusted *P* = 0.034, Figure 1H). This pathway describes a glucose fermentation process to produce acetate and lactate that is unique to bifidobacteria. Although most of the other pathways increased in the intervention group are also related to carbohydrate metabolism, they could not be attributed to a specific taxon and are therefore presumably reflective of an overall community dynamic. On the contrary, some pathways associated with DNA and lipid metabolism were decreased in the intervention group (Figure 1G). No significant impact of the intervention on the concentration of SCFA in feces was detected (Supplemental Table 6).

#### Plasma metabolites

**FIGURE 2:**
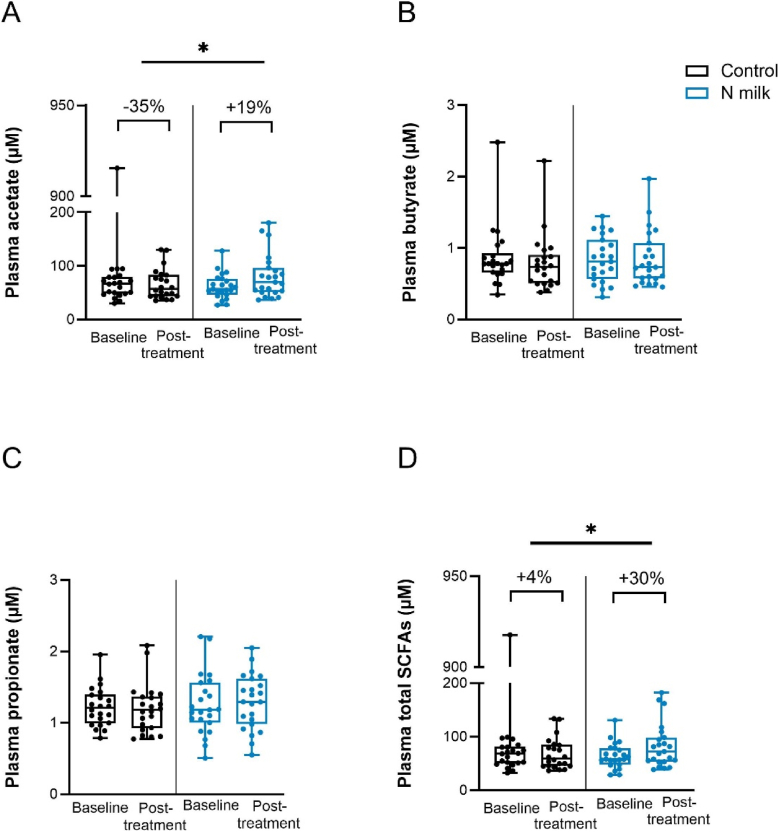
Impact of the intervention within and between groups in fasting plasma of acetate, butyrate, propionate, and total SCFA (*n* = 23) concentrations. Data expressed as mean, 95% CI, and individual datapoints. Data presented includes outliers. Percentage corresponds to the median change of posttreatment relative to baseline. ∗ represents a significant *P* value (≤ 0.05). Exact statistical results are reported in Supplemental Table 7. CI, confidence interval; SCFA, short-chain fatty acids.

Beyond the bifidogenic effect of N milk in the fecal microbiome, its effect on physiologically relevant compounds in plasma was explored, including 15 vitamins, 31 amino acids, ketone bodies, magnesium, and copper, as well as SCFA. N milk supplementation significantly increased fasting plasma acetate and total SCFA concentrations, compared with control (Figure 2), whereas other SCFA were not affected (Supplemental Table 7). Furthermore, a trend (*P* = 0.086) toward increased acetoacetate with N milk was observed, and no effect on other analytes. The bifidogenic effect of N milk, combined with the known capabilities of bifidobacteria to produce acetate and related compounds, and the observed effect on plasma metabolites, suggested a potential link between *Bifidobacterium* increase in the gut and host metabolism. Indeed, *Bifidobacterium* abundance correlated with acetoacetate (*r* = 0.168, *P* = 0.039), and there was a trend for positive correlation with 3-hydroxybutyrate (*r* = 0.173, *P* = 0.058) and acetate (*r* = 0.131, *P* = 0.077).

**FIGURE 3:**
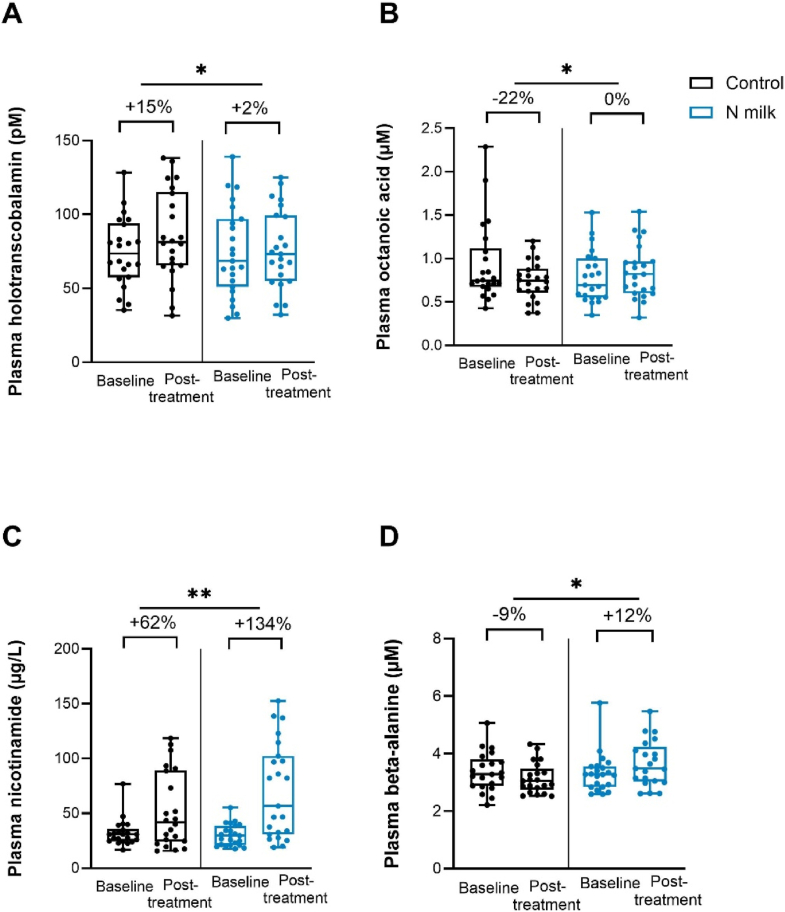
Impact of the intervention within and between groups in fasting plasma holotranscobalamin, nicotinamide, β-alanine, octanoic acid (*n* = 23). Data expressed as mean, 95% CI, and individual datapoints. Percentage corresponds to the median change of posttreatment relative to baseline. ∗ represents significant *P* ≤ 0.05, and ∗∗ *P* ≤ 0.01. Exact statistical results are reported in Supplemental Table 8. CI, confidence interval.

Fasting plasma holotranscobalamin, nicotinamide, β-alanine, and octanoic acid were significantly affected by N milk intake. Holotranscobalamin, the active form of vitamin B12, was decreased by –9.38 pM compared with control. Nicotinamide (form of vitamin B3), the amino acid β-alanine, and octanoic acid were increased by 15.29 μg/L, 0.53 μM, and 0.15 μM compared with control (Figure 3, Supplemental Table 7). Other compounds were not impacted (data not shown).

**FIGURE 4:**
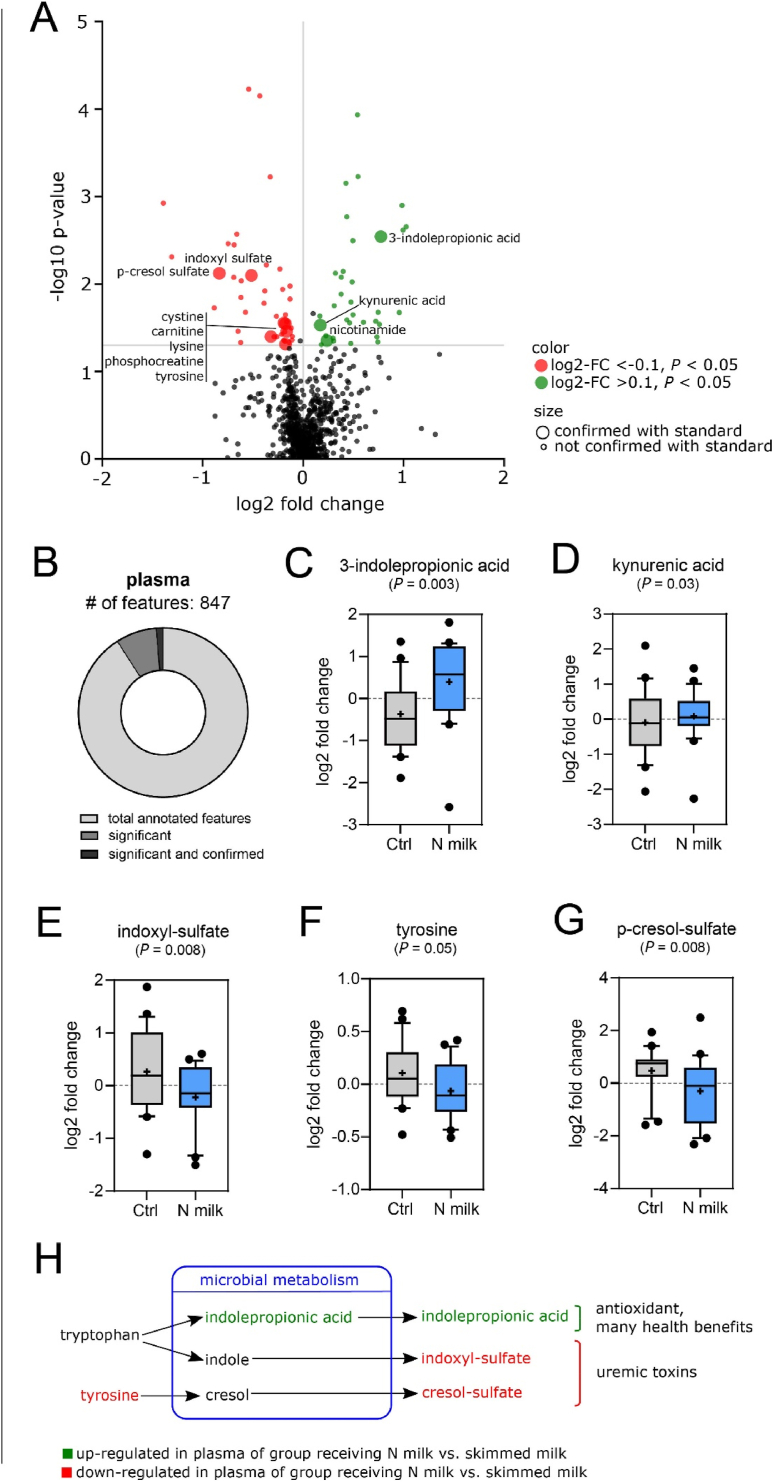
Impact of N milk on the plasma metabolome. (A) Volcano plot of metabolic features. The *x*-axis depicts the adjusted log2 difference between the N milk group and the lactose-free milk group at the end of the treatment. The *y*-axis shows the -log10 *P* values according to the lme model. (B) Pie chart of metabolic features analyzed according to their significance and confirmation. (C–G) Box plots of unadjusted log2 fold-changes from baseline over the treatments. *P* values were derived from the lme model. “+” indicates the mean for each group; whiskers represent the 10–90 percentiles. (H) Shift in microbial amino acid metabolism, which is induced by N milk in comparison with control.

To further explore potential effects of N milk, we deployed untargeted metabolomics for plasma samples to monitor semipolar and polar metabolites, such as amino acids and their derivatives, nucleotides, acyl-carnitines, and organic acids as a complement to the above-mentioned targeted measures. Of 847 metabolic features, which could be putatively annotated, 78 were differentially affected by the intervention, and among them, 10 were confirmed with authentic standards (Figure 4 A, B). An increase in nicotinamide was confirmed with the targeted method when comparing N milk to the control. Furthermore, we observed an effect of N milk on metabolic markers related to amino acid metabolism (Figure 4C–G), specifically in tryptophan metabolism. 3-indolepropionate and to a lesser extent kynurenic acid significantly increased on treatment with N milk in comparison to control [log2-difference of effect size of 0.77 (*P* = 0.003) and 0.17 (*P* = 0.03), respectively, Figure 4], whereas indoxyl-sulfate decreased (log2-difference of effect size –0.517, *P* = 0.008). In addition, tyrosine, and its microbial metabolite p-cresol sulfate, as well as lysine and the related metabolite carnitine were differentially affected by both interventions (Figure 4, Supplemental Figure 4). Among those, p-cresol sulfate showed the strongest change with a decrease in the N milk group compared with the control, where it increased having a differential treatment impact of –0.84 (*P* = 0.008). The induced shift in amino acid metabolism was further corroborated by distinctive changes in the cystine and arginine-related metabolite phosphocreatine (Supplemental Figure 4).

Overall, our data indicate that N milk intake affected bacterial amino acid metabolism, which was reflected in plasma metabolite levels. Yet, none of these metabolic features appeared significant after FDR adjustment.

#### Questionnaire-based endpoints

Overall, both products were well tolerated with no change in stool texture or appearance as assessed by the Bristol scale (baseline: 3.53; mean change with N milk = 0.01; control = –0.02, *P* = 0.97). Perceived stress State-Trait Anxiety Inventory (baseline: 33.2; mean change with N milk = –0.46; control = 0.907, *P* = 0.49), and 8 components of overall quality of life (SF-36) were similarly unaffected, with *P* values ranging from 0.05 to 0.76. Average GSRS scores remained <3 for all participants, with a minor increase observed with N milk (baseline: 1.43; mean change with N milk = 0.147; control = –0.038, *P* = 0.0048). The details of statistical outputs are in Supplemental Table 8.

#### Ex vivo batch fermentation

To better understand the direct impact of N milk in comparison with LF milk and GOS alone on colonic microbiome function, a short-term batch fermentation assay, preceded by a simulation of upper gastrointestinal tract digestion, was carried out using fecal samples from healthy adult donors.

#### Microbiome impact

**FIGURE 5:**
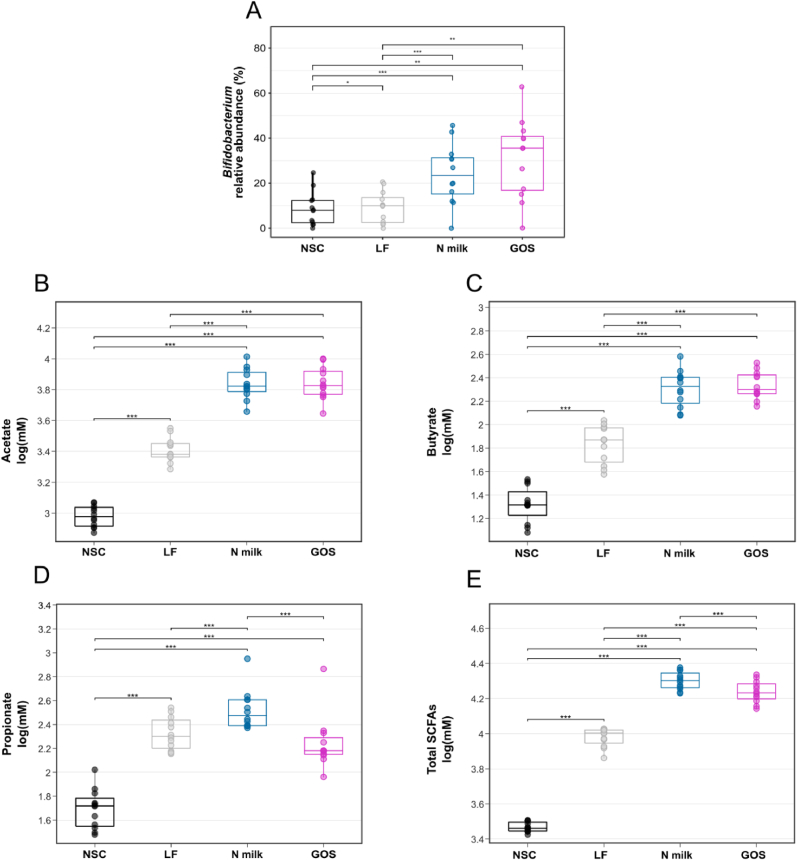
Short-term ex vivo batch culture fermentation. The impact after 24 h of ex vivo fermentation under the conditions control, lactose-free (LF) milk, N milk, and GOS on the (A) *Bifidobacterium* abundance and production of (B) acetate, (C) butyrate, (D) propionate, and (E) total SCFA. Statistical differences between groups are visualized via ∗ (0.01 < FDR-adjusted *P* value < 0.05), ∗∗ (0.001 < FDR-adjusted *P* value < 0.01), or ∗∗∗ (FDR-adjusted *P* value < 0.001). More details are in Supplemental Table 9. FDR, false discovery rate; GOS, galacto-oligosaccharides; NSC, no substrate control; SCFA, short-chain fatty acids.

After 24 h, a clear bifidogenic effect was observed in the N milk and GOS groups compared with no substrate control and LF groups (FDR-adjusted *P* values ranging from 0.00004 to 0.002, Figure 5A, Supplemental Table 9), mainly explained by an increase in the *Bifidobacterium adolescentis* relative abundance (FDR-adjusted *P* values ranging from 0.0004 to 0.03, Supplemental Figure 5, Supplemental Table 9). GOS and N milk groups showed a significantly lower Shannon diversity compared with control and LF groups (FDR-adjusted *P* = 0.001), with no significant difference in richness (Supplemental Table 9).

Although no significant difference in *Bifidobacterium* abundance was observed between N milk and GOS, several taxa, including *Akkermansia*, *Bacteroides*, *Dorea*, *Roseburia,* and *Sutterella* showed a significantly higher abundance in N milk compared with GOS; on the contrary, *Blautia* and *Faecalibacterium* showed a significantly higher abundance in GOS compared with N milk (FDR-adjusted *P* ≤ 0.005, Supplemental Table 9).

#### SCFA

Consistent with the bifidogenic effect of N milk and GOS, an increase in all 3 and in total SCFA production was observed compared with NSC group (FDR-adjusted *P* values ranging from 5.3 × 10^-5^ to 1.3 × 10^-12^, Figure 5B–E, Supplemental Table 10). Although N milk and GOS were equally efficient in promoting acetate and butyrate production (Figure 5B–C), only N milk increased propionate in comparison with LF milk (FDR-adjusted *P* = 0.0004, Figure 5D). The increase in total SCFA was significantly larger with N milk than with GOS alone (total SCFA FDR-adjusted *P* = 1.8 × 10^-6^) driven by the increase in propionate (Figure 5E, Supplemental Table 10). LF milk also increased SCFA production compared with NSC (total SCFA FDR-adjusted *P* = 1.8 × 10^-11^, Supplemental Table 10), despite a modest effect on the abundance of bifidobacteria (Figure 5A).

#### Other metabolites

**FIGURE 6:**
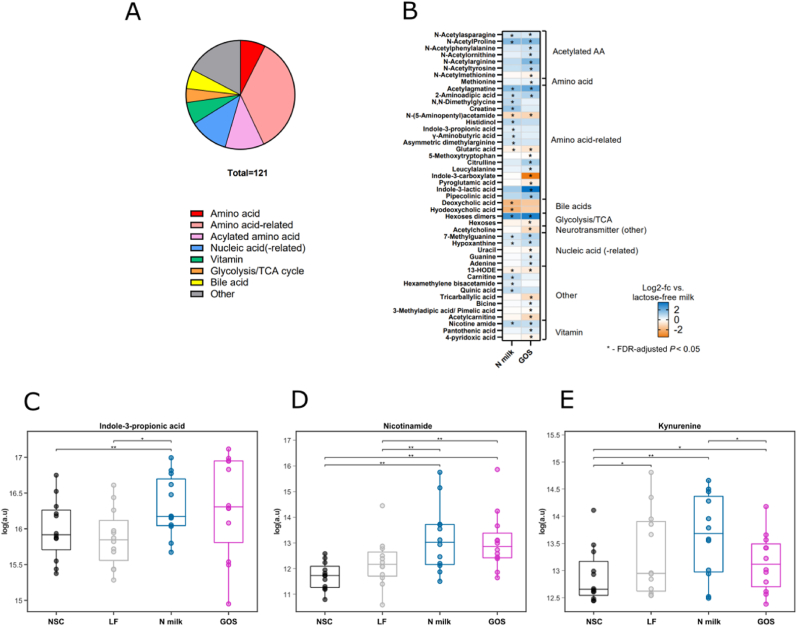
Semitargeted analysis of fermentation samples. (A) Metabolic classes detected in the fermentation supernatants. (B) Log2 fold changes of N milk and GOS-supplemented supernatants in comparison to fermentations with lactose-free milk after 24 h of fermentation. (C–E) Metabolite levels of 3-indolepropionate, nicotinamide, and kynurenine after 24 h of fermentation under different conditions: control (NSC), lactose-free (LF) milk, N milk, and GOS. ∗ represents significant *P* ≤ 0.05, and ∗∗*P* ≤ 0.01. More details are provided in Supplemental Table 10. GOS, galacto-oligosaccharides; NSC.

Overall microbial metabolism was further explored by semitargeted metabolomics, targeting 121 polar to semipolar metabolites (Figure 6A, Supplemental Table 10). Pairwise statistical analysis showed that 22 metabolites and 34 metabolites were significantly different between LF milk and N milk or LF milk and GOS, respectively. Although the changes induced by N milk or GOS tended to be in the same direction, the overlap in significantly affected metabolites was rather small (Figure 6B, Supplemental Table 10). Twelve amino acid metabolites were altered in N milk in comparison to LF milk including several acetylated amino acids and elevated indole-3-propionic acid (Figure 6C, Supplemental Table 10). The other tryptophan-related metabolite, kynurenine, also tended to increase in N milk in comparison with LF milk (FDR-adjusted *P* = 0.055) (Figure 6E, Supplemental Table 10). In addition, nicotinamide increased after the fermentation of N milk (or GOS alone) in comparison with LF milk (Figure 6D, Supplemental Table 10). On the other hand, metabolites such as secondary bile acids (deoxycholic acid and hyodeoxycholic acid), 13-HODE, glutaric acid, and N-(5-aminopentyl) acetamide increased with LF milk in comparison with the other conditions.

Overall, ex vivo fermentation supports the notion of a shift in microbial metabolism toward an elevation of 3-indolepropionate and nicotinamide, which were also found in the clinical study, and indicates the existence of additional metabolic adaptations induced by N milk.

## Discussion

We have assessed the short-term effects of N milk on the gut microbiome and related changes in health-related biomarkers in healthy volunteers in a randomized, double-blinded, controlled clinical trial. On the basis of the enzymatic conversion of lactose to GOS, N milk has a specific carbohydrate profile low in lactose and rich in GOS. Thus, the one and a half serving of N milk used in the study contained a substantial amount (9 g) of GOS. The clinical approach was complemented by an ex vivo fermentation experiment to gain insight into mechanisms of action, i.e., colonic fermentation of GOS embedded in milk matrix, resulting in increased microbial production of host-beneficial metabolites.

After a 2-wk intervention with N milk, we observed a highly significant increase in *Bifidobacterium* in the gut microbiome compared with the control group, with a 3-fold increase from 1.1% median baseline abundance (1.7% IQR) to 3.2% median abundance (4.1% IQR) after the intervention. The effects appear to be relatively large compared with previous studies with isolated GOS supplementation. Indeed, studies that used a similar measure of relative abundance reported a 1.3- to 2.2-fold increase in gut bifidobacteria [26,27,[53], [54], [55], [56], [57]]. Studies using the highest GOS doses of 10 g [56] and 11 g [57] per day reported effects in the higher range of 1.8- and 2.2-fold increase, respectively. The only study reporting a much higher (19-fold) response was characterized by a very low baseline bifidobacteria relative abundance of 0.09% [58]. In the above-mentioned studies, the baseline bifidobacteria abundance ranged from 3.8 to a surprisingly high abundance of 30% in adults [53]. In our study, a relatively low median baseline abundance of 1.1% was observed, suggesting that a more pronounced effect may be achieved in these circumstances. Overall, we hypothesize that the high efficacy of our intervention might have been linked to a robust dose of GOS of 9 g, and a relatively low baseline relative abundance of bifidobacteria among the participants, potentially linked to low fiber intake in the study population.

The use of shotgun metagenomics allowed us to evaluate the effect of our intervention on individual species. Interestingly, all 5 bifidobacteria species detected with sufficient prevalence, namely *B. longum*, *B*. *adolescentis*, *B*. *catenulatum*, *B. pseudocatenulatum*, and *B. bifidum,* benefited from the addition of GOS. This suggests that the presence of any bifidobacteria species at baseline may be sufficient to achieve a response in overall bifidobacteria abundance. However, the small size of our study does not permit further evaluation of whether specific species are more responsive to the intervention.

Our study showed that bifidobacteria levels returned to baseline after a 2-wk washout period, underscoring the transient nature of microbiome modulation by our intervention and suggesting that continuous or repeated intake may be necessary to maintain its bifidogenic effect. From a mechanistic perspective, GOS resists hydrolysis by human digestive enzymes due to its β-glycosidic linkages, allowing it to reach the colon intact. There, the bifidogenic effect of GOS can be attributed to the metabolic and enzymatic processes of bifidobacteria, which allow for preferential utilization of the prebiotic. Indeed, bifidobacteria possess β-galactosidase enzymes, enabling the hydrolysis of GOS into monosaccharides [59]. Moreover, bifidobacteria are equipped with specific carbohydrate transport systems, such as the LacS symporter, which facilitate the uptake of GOS into the bacterial cell [60].

We observed that the bifidogenic effect of N milk was matched by a shift in microbiome functional capacity, notably an increase in carbohydrate metabolism pathways and a decrease in DNA and lipid metabolism pathways, suggesting a possible trade-off driven by bifidobacteria within the microbiome’s metabolic resources. Concomitant with the increase of bifidobacteria in the intervention group, we observed a significant increase of the *Bifidobacterium* shunt pathway, which ensures high competitiveness of bifidobacteria via efficient energy production [59]. Indeed, this pathway metabolizes GOS and its byproducts, which leads to the production of SCFA, mostly acetate and lactate. It was previously shown that the carbohydrate substrate modifies the flux distribution of this central carbon metabolism, which then leads to a modified SCFA ratio distribution [61].

Beyond the substantial increase in bifidobacteria and corresponding SCFA production pathways, we observed a considerable increase in plasma acetate in study participants. The observed levels at the end of the intervention were comparable to levels previously achieved via acetate infusions in the distal colon [62], which were accompanied by effects on fat oxidation and gut hormone secretion. Compared with other colonic regions, the distal colon represents a specific region in which acetate absorption can partly by-pass liver utilization and be diverted and used by other metabolically active tissues such as muscle, adipose tissue, and pancreas. The distal colon also has a greater expression of free fatty acid receptor in the enteroendocrine L-cells, which, if stimulated with SCFA, can lead to the secretion of anorectic gut hormones with roles in glucose metabolism [63]. Boosting acetate production by bifidobacteria residing in the distal colon could thus be a highly efficient way to impact circulating plasma acetate concentrations.

Consistent with our findings, a previous study showed an increase in bifidobacteria and a decrease in inflammatory and lipid profiles after GOS supplementation for 12 wk in overweight adults. However, plasma SCFA were not measured in the study [27]. In contrast, a study using the same design in prediabetic adults with obesity found no changes in plasma SCFA after GOS intervention despite having observed a significant increase in bifidobacteria [64]. Outcome inconsistencies between studies may be due to factors such as demographic or intervention differences. In addition, embedding GOS in the milk matrix may facilitate SCFA production, as suggested by ex vivo experiments.

In our study, N milk supplementation increased plasma concentration of several compounds linked to energy metabolism, including β-alanine, nicotinamide, octanoic acid, and a trend for an increase in acetoacetate. This indicates that our intervention combining GOS with very low mono- and disaccharide content may have altered fat metabolism via alternative mechanisms. Moreover, acetate may have served as a precursor for both octanoic acid and the ketone body acetoacetate in the liver to be further used for energy production.

The increase of nicotinamide that we observed could be explained by genomic enrichment of the bacterial NAD salvage pathway I activation in the microbiota. Moreover, nicotinamide is a known precursor of NAD, which has multiple functions in energy metabolism, including the Kreb’s cycle, glycolysis, and fat oxidation. β-Alanine has been associated with muscle fatigue prevention as a precursor of carnosine, a compound that helps buffer acid in the muscles. Together, there are indications that N milk intake led to adaptations in energy metabolism, although the precise mechanisms remain to be elucidated. Notably, holotranscobalamin, the active form of vitamin B12, showed a reduction with N milk supplementation. However, this effect appeared minor; it was smaller than the variations observed in healthy lacto-vegetarian women over 2–6 wk following their usual lifestyle [65]. Further exploration of plasma metabolites using an untargeted approach demonstrated that the intake of N milk promoted a subtle, yet consistent shift in amino acid metabolism. Microbial tryptophan metabolism was shifted toward increased 3-indolepropionate and, to a lesser extent, higher kynurenic acid concentrations, similar to findings related to fiber intake [66]. Concomitantly, the uremic toxins indoxyl-sulfate and the tyrosine-derived p-cresol-sulfate differentially decreased. Although a shift in tryptophan metabolism has been reported with dietary fiber from various sources [67,68], our data suggest that N milk, combining GOS with low lactose, embedded in a milk matrix, alone might be sufficient to achieve this. Low concentrations of 3-indolepropionate have been associated with various health conditions, such as neurodegeneration, inflammation, and cardiovascular disease [[21], [22], [23],[69], [70], [71]]. In addition, preclinical intervention studies have shown a positive effect of indole-3-propionate on the gut barrier, gut–organ interactions (e.g., gut–brain-axis, gut–liver-axis), and immune function [[21], [22], [23],^69^]. The simultaneous reduction of the uremic toxins indoxyl-sulfate and p-cresol-sulfate, which might relate to an increased burden for the kidney and oxidative stress [72]. These shifts are indicative that sustained intake of N milk may lead to long-term benefits.

Despite a large increase in bifidobacteria and pathways to produce acetate, we have not observed increased acetate or overall SCFA concentrations in the stools. Although some studies in infants show a decrease in pH and a modest increase in fecal acetate linked with increased bifidobacteria [73,74], in adults, the results are much more variable. Many microbially produced metabolites are readily absorbed, rapidly utilized by other bacteria, or otherwise degraded before being excreted in stool. Therefore, systemic rather than fecal SCFA concentrations reflect intestinal production [75]. Interestingly, some studies observed that adults suffering from health conditions linked to increased gut permeability and presumably deficient absorption [76] had increased SCFA in the feces compared with healthy adults.

Our ex vivo fermentation experiment allowed us to directly evaluate the metabolic activity of colonic bacteria and resulting metabolites before absorption by the host, providing a link between the observed effect on microbiome and plasma metabolome. As expected, ex vivo fermentation with N milk resulted in an increase of bifidobacteria and abundant production of SCFA, leading us to hypothesize that colonic acetate production was induced by N milk in the clinical study, but its rapid absorption precluded detection in fecal samples. The transient presence of microbially produced acetate in the colon would need to be confirmed to better understand if it can locally exert already known benefits, such as strengthening of the gut barrier and protection from enteropathogenic infections [77]. Regarding other metabolites, a shift in amino acid metabolism, in particular tryptophan (indole-3-propionic acid and kynurenine) and nicotinamide, was observed, corroborating the relevance of a similar subtle shift observed in the clinical study.

Several differences between N milk and GOS effects were observed regarding the produced metabolites. Notably, total SCFA production, driven by propionate, was higher with N milk than with GOS alone. In addition, some increase in SCFA production was observed with LF milk alone. Even though milk should be largely digested in the first phase of the experiment, this indicates that even small amounts of milk digesta reaching the colon exert an independent or synergistic with GOS effect on colonic microbiome in vivo.

No clinically relevant differences were observed in questionnaire-based endpoints. There is some indication that large doses of GOS, particularly in individuals with low fiber intake, could lead to low-level transient increase in gastrointestinal discomfort, which subsequently subsides following the adaptation of gut microbiome [7]. Despite a large dose of GOS and the baseline fiber intake of our participants below recommendations, we have observed only a minor increase in GSRS with N milk, and the overall score remained below the threshold of 3 in both groups, indicating a lack of clinical significance. A small increase in GSRS score was observed with only 2 g of GOS [53] in healthy women, indicating that other factors, either linked to participant characteristics, details of study design, or features of the formulation, are likely to modulate the intensity of GI symptoms, thus meriting further research.

Strengths of our study include a complementary clinical and ex vivo approach and in-depth analysis of clinical and ex vivo samples using complementary molecular approaches. Limitations include a relatively small sample size as well as a short duration, precluding observing chronic effects of the detected shift in the host metabolic signature. More detailed assessment of dietary intake than was feasible with food frequency questionnaire would bring more insights into potential modifications of dietary intake as previously reported [53].

To our knowledge, this is the first time N milk was tested with a clinical approach; thus, further clinical studies are required to confirm the observed effects. At present, our conclusions are only fully applicable to healthy adults, even though the underlying mechanisms, such as a boost of bifidobacteria with GOS, are known to occur in pediatric and elderly populations [27,78]. Similarly, our conclusions are limited to N milk, and GOS embedded therein. GOS preparations vary somewhat in terms of molecular structure [24]; however, the functional impact of these differences is not clear [79].

In conclusion, present findings indicate that consumption of low-lactose, GOS-rich milk for 2 wk in healthy adults resulted in increased gut bifidobacteria and their metabolic activity, resulting in a shift in plasma metabolites toward a profile linked with beneficial immune and metabolic health outcomes. The effects of N milk appear to reflect the established positive effects of GOS and may potentially enhance them due to embedding within the milk matrix.

N milk, with 9 g of GOS per 1.5 serving, may present a promising solution to encourage the consumption of both dairy and fiber, whose intake often falls short of recommended levels in various populations. Both dairy and fiber intake are linked to positive health outcomes, and in N milk, these 2 essential components are conveniently combined into a single product.

## Author contributions

The authors’ responsibilities were as follows – LS: analyzed data, performed statistical analysis, and wrote manuscript; AC-M, J-PG: analyzed data, wrote manuscript; CLB: designed research, conducted research, and performed statistical analysis; AL: performed statistical analysis; CJC: designed research; FF, AF: conducted research; MPG: analyzed data and performed statistical analysis; SC: conducted research, analyzed data, and wrote the manuscript; OS: designed the research, wrote the manuscript, and had primary responsibility for final content; and all authors: read and approved the manuscript.

## Data availability

Data described in the manuscript are not available due to data privacy and ethical restrictions.

## Funding

Funded by Société des Produits Nestlé SA.

## Conflict of interest

All authors are, or were, employees of Société des Produits Nestlé when this work was conducted. Patents related to findings described in this study were filed with OS, LS, J-PG, MPG, SC, and AC-M listed as inventors.

## References

[1] McKeown N.M., Fahey G.C., Slavin J., van der Kamp J.W. (2022). Fibre intake for optimal health: how can healthcare professionals support people to reach dietary recommendations?. BMJ.

[2] McGill C.R., Fulgoni V.L., Devareddy L. (2015). Ten-year trends in fiber and whole grain intakes and food sources for the United States population: National Health and Nutrition Examination Survey 2001-2010. Nutrients.

[3] Gressier M., Frost G. (2022). Minor changes in fibre intake in the UK population between 2008/2009 and 2016/2017. Eur. J. Clin. Nutr..

[4] Yu D., Zhao L., Zhao W. (2020). Status and trends in consumption of grains and dietary fiber among Chinese adults (1982-2015). Nutr. Rev..

[5] Collaborators GBDD (2019). Health effects of dietary risks in 195 countries, 1990–2017: a systematic analysis for the Global Burden of Disease Study 2017. Lancet.

[6] Mohr P., Quinn S., Morell M., Topping D. (2010). Engagement with dietary fibre and receptiveness to resistant starch in Australia. Public Health Nutr.

[7] Borkoles E., Krastins D., van der Pols J.C., Sims P., Polman R. (2022). Short-term effect of additional daily dietary fibre intake on appetite, satiety, gastrointestinal comfort, acceptability, and feasibility. Nutrients.

[8] McDermott A.J., Huffnagle G.B. (2014). The microbiome and regulation of mucosal immunity. Immunology.

[9] Holmes E., Li J.V., Athanasiou T., Ashrafian H., Nicholson J.K. (2011). Understanding the role of gut microbiome-host metabolic signal disruption in health and disease. Trends Microbiol.

[10] Udayappan S.D., Hartstra A.V., Dallinga-Thie G.M., Nieuwdorp M. (2014). Intestinal microbiota and faecal transplantation as treatment modality for insulin resistance and type 2 diabetes mellitus. Clin. Exp. Immunol..

[11] de Groot P., Nikolic T., Pellegrini S., Sordi V., Imangaliyev S., Rampanelli E. (2021). Faecal microbiota transplantation halts progression of human new-onset type 1 diabetes in a randomised controlled trial. Gut.

[12] Suskind D.L., Brittnacher M.J., Wahbeh G., Shaffer M.L., Hayden H.S., Qin X. (2015). Fecal microbial transplant effect on clinical outcomes and fecal microbiome in active Crohn's disease. Inflamm. Bowel Dis..

[13] Blaak E.E., Canfora E.E., Theis S., Frost G., Groen A.K., Mithieux G. (2020). Short chain fatty acids in human gut and metabolic health. Benef. Microbes..

[14] Gibson G.R., Hutkins R., Sanders M.E., Prescott S.L., Reimer R.A., Salminen S.J. (2017). Expert consensus document: the International Scientific Association for Probiotics and Prebiotics (ISAPP) consensus statement on the definition and scope of prebiotics. Nat. Rev. Gastroenterol. Hepatol..

[15] Frampton J., Murphy K.G., Frost G., Chambers E.S. (2020). Short-chain fatty acids as potential regulators of skeletal muscle metabolism and function. Nat. Metab..

[16] Pingitore A., Chambers E.S., Hill T., Maldonado I.R., Liu B., Bewick G. (2017). The diet-derived short chain fatty acid propionate improves beta-cell function in humans and stimulates insulin secretion from human islets in vitro. Diabetes Obes. Metab..

[17] Frost G., Sleeth M.L., Sahuri-Arisoylu M., Lizarbe B., Cerdan S., Brody L. (2014). The short-chain fatty acid acetate reduces appetite via a central homeostatic mechanism. Nat. Commun..

[18] Kim C.H. (2023). Complex regulatory effects of gut microbial short-chain fatty acids on immune tolerance and autoimmunity. Cell Mol. Immunol..

[19] Ye X., Li H., Anjum K., Zhong X., Miao S., Zheng G. (2022). Dual role of indoles derived from intestinal microbiota on human health. Front. Immunol..

[20] Xue C., Li G., Zheng Q., Gu X., Shi Q., Su Y. (2023). Tryptophan metabolism in health and disease. Cell Metab..

[21] Roager H.M., Licht T.R. (2018). Microbial tryptophan catabolites in health and disease. Nat. Commun..

[22] Konopelski P., Mogilnicka I. (2022). Biological effects of indole-3-propionic acid, a gut microbiota-derived metabolite, and its precursor tryptophan in mammals' health and disease. Int. J. Mol. Sci..

[23] Negatu D.A., Gengenbacher M., Dartois V., Dick T. (2020). Indole propionic acid, an unusual antibiotic produced by the gut microbiota, with anti-inflammatory and antioxidant properties. Front. Microbiol..

[24] van Leeuwen S.S., Kuipers B.J., Dijkhuizen L., Kamerling J.P. (2016). Comparative structural characterization of 7 commercial galacto-oligosaccharide (GOS) products. Carbohydr. Res..

[25] Gonai M., Shigehisa A., Kigawa I., Kurasaki K., Chonan O., Matsuki T. (2017). Galacto-oligosaccharides ameliorate dysbiotic bifidobacteriaceae decline in Japanese patients with type 2 diabetes. Benef. Microbes..

[26] Walton G.E., van den Heuvel E.G., Kosters M.H., Rastall R.A., Tuohy K.M., Gibson G.R. (2012). A randomised crossover study investigating the effects of galacto-oligosaccharides on the faecal microbiota in men and women over 50 years of age. Br. J. Nutr..

[27] Vulevic J., Juric A., Walton G.E., Claus S.P., Tzortzis G., Toward R.E. (2015). Influence of galacto-oligosaccharide mixture (B-GOS) on gut microbiota, immune parameters and metabonomics in elderly persons. Br. J. Nutr..

[28] Vulevic J., Drakoularakou A., Yaqoob P., Tzortzis G., Gibson G.R. (2008). Modulation of the fecal microflora profile and immune function by a novel trans-galactooligosaccharide mixture (B-GOS) in healthy elderly volunteers. Am. J. Clin. Nutr..

[29] Boger M., van Leeuwen S.S., Lammerts van Bueren A., Dijkhuizen L. (2019). Structural identity of galactooligosaccharide molecules selectively utilized by single cultures of probiotic bacterial strains. J. Agric. Food Chem..

[30] van Leeuwen S.S., Te Poele E.M., Chatziioannou A.C., Benjamins E., Haandrikman A., Dijkhuizen L. (2020). Goat milk oligosaccharides: their diversity, quantity, and functional properties in comparison to human milk oligosaccharides. J. Agric. Food Chem..

[31] Vénica C.I., Solís M.A., Senovieski M.L., Vélez M.A., Spotti M.J., Giménez P. (2025). Combining strategies for the development of a potentially functional yogurt: structural, physicochemical, and microbiological characterization. Food Bioprocess Technol.

[32] Pereira P.C. (2014). Milk nutritional composition and its role in human health. Nutrition.

[33] Auestad N., Layman D.K. (2021). Dairy bioactive proteins and peptides: a narrative review. Nutr. Rev..

[34] Stewart H., Kuchler F. (2022). https://www.ers.usda.gov/amber-waves/2022/june/fluid-milk-consumption-continues-downward-trend-proving-difficult-to-reverse/.

[35] Aili L., Jie Z., Xueting H., Zehua J., Bowen Y., Sijia Y. (2023). Health implication of lactose intolerance and updates on its dietary management. Int. Dairy J..

[36] Lewis S.J., Heaton K.W. (1997). Stool form scale as a useful guide to intestinal transit time. Scand. J. Gastroenterol..

[37] Svedlund J., Sjodin I., Dotevall G. (1988). GSRS—a clinical rating scale for gastrointestinal symptoms in patients with irritable bowel syndrome and peptic ulcer disease. Dig. Dis. Sci..

[38] Spielberger C.D., Gorsuch R.L., Lushene R.E., Vagg P.R., Jacobs G. (1983).

[39] Stull V.J., Finer E., Bergmans R.S., Febvre H.P., Longhurst C., Manter D.K. (2018). Impact of edible cricket consumption on gut microbiota in healthy adults, a double-blind, randomized crossover trial. Sci. Rep..

[40] Blanco-Miguez A., Beghini F., Cumbo F., McIver L.J., Thompson K.N., Zolfo M. (2023). Extending and improving metagenomic taxonomic profiling with uncharacterized species with MetaPhlAn 4. Nat. Biotechnol..

[41] Beghini F., McIver L.J., Blanco-Miguez A., Dubois L., Asnicar F., Maharjan S. (2021). Integrating taxonomic, functional, and strain-level profiling of diverse microbial communities with bioBakery 3. Elife.

[42] Valdivia-Garcia M.A., Chappell K.E., Camuzeaux S., Olmo-Garcia L., van der Sluis V.H., Radhakrishnan S.T. (2022). Improved quantitation of short-chain carboxylic acids in human biofluids using 3-nitrophenylhydrazine derivatization and liquid chromatography with tandem mass spectrometry (LC-MS/MS). J. Pharm. Biomed. Anal..

[43] Van den Abbeele P., Deyaert S., Thabuis C., Perreau C., Bajic D., Wintergerst E. (2023). Bridging preclinical and clinical gut microbiota research using the ex vivo SIFR(R) technology. Front. Microbiol..

[44] De Weirdt R., Possemiers S., Vermeulen G., Moerdijk-Poortvliet T.C., Boschker H.T., Verstraete W. (2010). Human faecal microbiota display variable patterns of glycerol metabolism. FEMS Microbiol. Ecol..

[45] Callahan B.J., McMurdie P.J., Rosen M.J., Han A.W., Johnson A.J., Holmes S.P. (2016). DADA2: high-resolution sample inference from Illumina amplicon data. Nat. Methods..

[46] Bolyen E., Rideout J.R., Dillon M.R., Bokulich N.A., Abnet C.C., Al-Ghalith G.A. (2019). Reproducible, interactive, scalable and extensible microbiome data science using QIIME 2. Nat. Biotechnol..

[47] Bokulich N.A., Kaehler B.D., Rideout J.R., Dillon M., Bolyen E., Knight R. (2018). Optimizing taxonomic classification of marker-gene amplicon sequences with QIIME 2's q2-feature-classifier plugin. Microbiome.

[48] McDonald D., Price M.N., Goodrich J., Nawrocki E.P., DeSantis T.Z., Probst A. (2012). An improved Greengenes taxonomy with explicit ranks for ecological and evolutionary analyses of bacteria and archaea. ISME J.

[49] Kieser S., Sarker S.A., Sakwinska O., Foata F., Sultana S., Khan Z. (2018). Bangladeshi children with acute diarrhoea show faecal microbiomes with increased Streptococcus abundance, irrespective of diarrhoea aetiology. Environ. Microbiol..

[50] Doneanu C.E., Chen W., Mazzeo J.R. (2011). UPLC/MS monitoring of water-soluble vitamin Bs in cell culture media in minutes. Water Appl. Note.

[51] Adams K.J., Pratt B., Bose N., Dubois L.G., John-Williams L. St., Perrott K.M. (2020). Skyline for small molecules: a unifying software package for quantitative metabolomics. J. Proteome Res..

[52] Dwan K., Li T., Altman D.G., Elbourne D. (2019). CONSORT 2010 statement: extension to randomised crossover trials. BMJ.

[53] Looijesteijn E., Schoemaker M.H., van den Belt M., Hester E.R., Kortman G.A.M., Viskaal-van Dongen M. (2024). A double-blind intervention trial in healthy women demonstrates the beneficial impact on Bifidobacterium with low dosages of prebiotic galacto-oligosaccharides. Front. Nutr..

[54] Johnstone N., Dart S., Knytl P., Nauta A., Hart K., Cohen Kadosh K. (2021). Nutrient intake and gut microbial genera changes after a 4-week placebo controlled galacto-oligosaccharides intervention in young females. Nutrients.

[55] Krumbeck J.A., Rasmussen H.E., Hutkins R.W., Clarke J., Shawron K., Keshavarzian A. (2018). Probiotic Bifidobacterium strains and galactooligosaccharides improve intestinal barrier function in obese adults but show no synergism when used together as synbiotics. Microbiome.

[56] Ito M., Deguchi Y., Miyamori A., Matsumoto K., Kikuchi H., Matsumoto K. (1990). Effects of administration of galactooligosaccharides on the human faecal microflora, stool weight and abdominal sensation. Microb. Ecol. Health Dis..

[57] Schoemaker M.H., Hageman J.H.J., Ten Haaf D., Hartog A., Scholtens P., Boekhorst J. (2022). Prebiotic galacto-oligosaccharides impact stool frequency and fecal microbiota in self-reported constipated adults: a randomized clinical trial. Nutrients.

[58] Bouhnik Y., Flourie B., D'Agay-Abensour L., Pochart P., Gramet G., Durand M. (1997). Administration of transgalacto-oligosaccharides increases fecal bifidobacteria and modifies colonic fermentation metabolism in healthy humans. J. Nutr..

[59] Pokusaeva K., Fitzgerald G.F., van Sinderen D. (2011). Carbohydrate metabolism in bifidobacteria. Genes Nutr.

[60] Akiyama T., Kimura K., Hatano H. (2015). Diverse galactooligosaccharides consumption by bifidobacteria: implications of β-galactosidase—LacS operon. Biosci. Biotechnol. Biochem..

[61] Duboux S., Pruvost S., Joyce C., Bogicevic B., Muller J.A., Mercenier A. (2023). The pleiotropic effects of carbohydrate-mediated growth rate modifications in Bifidobacterium longum NCC 2705. Microorganisms.

[62] van der Beek C.M., Canfora E.E., Lenaerts K., Troost F.J., Damink S., Holst J.J. (2016). Distal, not proximal, colonic acetate infusions promote fat oxidation and improve metabolic markers in overweight/obese men. Clin. Sci. (Lond)..

[63] Larraufie P., Martin-Gallausiaux C., Lapaque N., Dore J., Gribble F.M., Reimann F. (2018). SCFAs strongly stimulate PYY production in human enteroendocrine cells. Sci. Rep..

[64] Canfora E.E., van der Beek C.M., Jocken J.W.E., Goossens G.H., Holst J.J., Olde Damink S.W.M. (2017). Colonic infusions of short-chain fatty acid mixtures promote energy metabolism in overweight/obese men: a randomized crossover trial. Sci. Rep..

[65] Naurath H.J., Joosten E., Riezler R., Stabler S.P., Allen R.H., Lindenbaum J. (1995). Effects of vitamin B12, folate, and vitamin B6 supplements in elderly people with normal serum vitamin concentrations. Lancet.

[66] Sinha A.K., Laursen M.F., Brinck J.E., Rybtke M.L., Hjorne A.P., Prochazkova N. (2024). Dietary fibre directs microbial tryptophan metabolism via metabolic interactions in the gut microbiota. Nat. Microbiol..

[67] Tuomainen M., Lindstrom J., Lehtonen M., Auriola S., Pihlajamaki J., Peltonen M. (2018). Associations of serum indolepropionic acid, a gut microbiota metabolite, with type 2 diabetes and low-grade inflammation in high-risk individuals. Nutr. Diabetes.

[68] Qi Q., Li J., Yu B., Moon J.Y., Chai J.C., Merino J. (2022). Host and gut microbial tryptophan metabolism and type 2 diabetes: an integrative analysis of host genetics, diet, gut microbiome and circulating metabolites in cohort studies. Gut.

[69] Jiang H., Chen C., Gao J. (2022). Extensive summary of the important roles of indole propionic acid, a gut microbial metabolite in host health and disease. Nutrients.

[70] Niu B., Pan T., Xiao Y., Wang H., Zhu J., Tian F. (2025). The therapeutic potential of dietary intervention: based on the mechanism of a tryptophan derivative-indole propionic acid on metabolic disorders. Crit. Rev. Food Sci. Nutr..

[71] Kim C.S., Jung S., Hwang G.S., Shin D.M. (2023). Gut microbiota indole-3-propionic acid mediates neuroprotective effect of probiotic consumption in healthy elderly: a randomized, double-blind, placebo-controlled, multicenter trial and in vitro study. Clin. Nutr..

[72] Watanabe H., Miyamoto Y., Enoki Y., Ishima Y., Kadowaki D., Kotani S. (2015). p-Cresyl sulfate, a uremic toxin, causes vascular endothelial and smooth muscle cell damages by inducing oxidative stress. Pharmacol. Res. Perspect..

[73] Capeding M.R.Z., Phee L.C.M., Ming C., Noti M., Vidal K., Le Carrou G. (2023). Safety, efficacy, and impact on gut microbial ecology of a Bifidobacterium longum subspecies infantis LMG11588 supplementation in healthy term infants: a randomized, double-blind, controlled trial in the Philippines. Front. Nutr..

[74] Bosheva M., Tokodi I., Krasnow A., Pedersen H.K., Lukjancenko O., Eklund A.C. (2022). Infant formula with a specific blend of five human milk oligosaccharides drives the gut microbiota development and improves gut maturation markers: a randomized controlled trial. Front. Nutr..

[75] Kirschner S.K., Engelen M.P., Haas P., Have G., Thaden J., Deutz N.E. (2023). Systemic but not fecal short-chain fatty acid (SCFA) concentrations reflect intestinal SCFA production as assessed by novel stable isotope method. Clin Nutr ESPEN.

[76] de la Cuesta-Zuluaga J., Mueller N.T., Alvarez-Quintero R., Velasquez-Mejia E.P., Sierra J.A., Corrales-Agudelo V. (2018). Higher fecal short-chain fatty acid levels are associated with gut microbiome dysbiosis, obesity, hypertension, and cardiometabolic disease risk factors. Nutrients.

[77] Fukuda, Toh H., Hase K., Oshima K., Nakanishi Y., Yoshimura K. (2011). Bifidobacteria can protect from enteropathogenic infection through production of acetate. Nature.

[78] Arslanoglu S., Moro G.E., Boehm G. (2007). Early supplementation of prebiotic oligosaccharides protects formula-fed infants against infections during the first 6 months of life. J. Nutr..

[79] Cramer J.F., Kjaer K.H., Yde C.C., Wichmann J., Jensen H.M., Kortman G.A.M. (2025). β-galactosidase from Bifidobacterium bifidum for improved in situ synthesis of GOS-oligomers with prebiotic effects. Int. Dairy J..

