# Supplementary material for: The Impact of Low-Lactose, High Galacto-Oligosaccharides Milk on Gut Microbiome and Plasma Metabolome in Healthy Adults: A Randomized, Double-Blind, Controlled Clinical Trial Complemented by Ex Vivo Experiments
Source: Curr Dev Nutr. 2025 Jul 24;9(9):107506. doi: 10.1016/j.cdnut.2025.107506 (PMC12405628; doi:10.1016/j.cdnut.2025.107506)
Supplement: Multimedia component 1 [file mmc1.docx]

# The impact of low-lactose, high galacto-oligosaccharides (GOS) milk on gut microbiome and plasma metabolome in healthy adults: A randomized, double-blind, controlled clinical trial complemented by ex vivo experiments

# Siegwald et al

# **Supplementary Methods**

#### **Clinical study: Inclusion and Exclusion Criteria**

Subjects’ inclusion criteria included: age 18-55 years, overall good health, both male and female, 18.5 ≤ BMI ≤ 29.9 kg/m2, be able to understand and to sign a written informed consent. Subjects’ exclusion criteria included: known chronic diseases or conditions; known food allergy and intolerance; habitually, have < 5 spontaneous bowel movements on average per week; chronic or recurrent diarrhea; prior gastrointestinal surgery; received systemic antiviral/antibacterial/antifungal therapy during the 3 months prior to study; drugs or supplements that are known to alter gut function or microflora during the 4 weeks prior to study; anti-hyperlipidaemic, antihypertensive medications and/or anticoagulant agents; currently participating in another interventional clinical trial; alcohol intake > 2 servings per day; chronic substance abuse; changing diet patterns due to travelling; food restrictions (e.g. vegan); restrictive-diets; artificially sweetened beverage intake >1000 ml/day; pregnant and lactating women; occurrence of fever episodes, infection, or vaccination during 14 days prior to the study.

#### **Clinical study: Randomization and Blinding**

The two sequences (interventional-control and control-interventional) were defined in the protocol and entered as parameters in the Medidata Rave RTSM software (<https://www.medidata.com/en/clinical-trial-products/clinical-data-management/rtsm/>).

#### **Clinical study: Sample collection**

Blood samples were collected in EDTA-K3 tubes (Sarstedt, Nümbrecht, Germany). For the serum, a tube without anti-coagulant was used. Blood samples were inverted 5 times after collection and stored at room temperature (serum) on ice for 30-45 minutes. Plasma and serum were obtained by centrifugation of whole blood at 2500 x g for 10 min at 4°C and aliquoted in 0.7 ml FluidX tubes and stored at -80°C for further analysis.

Each participant was instructed to collect one stool sample either on the day before or on the same day of the blood sampling visit. The stool sample was kept frozen in domestic freezer (-20°C) overnight, transported to the lab under - 20°C, and further stored at -80°C until analysis.

#### **Clinical study: Fecal short chain fatty acid analysis by GC-MS**

The samples (calibration curves, quality control and study samples) preparation, involving a derivatization step, was automated and carried out on a Microlab Star M liquid handler (Hamilton). Briefly, fecal water (containing orthophosphoric acid at 0.1% with internal standards) were deproteinized with 5-sulfosalicylic acid and extracted with chloroform before being derivatized with tert-butyldimethylsilylimidazole (TBDMSIM). Finally, the samples were diluted with chloroform before being analyzed by a gas chromatography-mass spectrometer (GC-MS) on a DB-5MS UI column, 0.25 µm, 30m x 0.25 mm (Agilent). Data were acquired in selected ion monitoring (SIM) acquisition mode. Chromatographic peaks were integrated using Openlab CDS2 software (Agilent).

#### **Clinical study: Plasma short chain fatty acid analysis by LC-MSMS**

Plasma short chain fatty acid (SCFA) analysis was carried out using an Acquity *I*-class UPLC system (Waters) coupled to a tandem mass spectrometer (Sciex 6500+). 40 μL of plasma sample were deproteinized and derivatized with 3-nitrophenylhydrazine (3-NPH) according to Valdivia-Garcia *et al.* (1). Derivatized compounds (lactic, acetic, propionic, butyric, valeric, isobutyric, isovaleric, 3-methylvaleric, hexanoic, octanoic, decanoic, 3-hydroxybutyric acids, and acetoacetate) were detected and quantified in negative mode using multiple reaction monitoring (MRM) transitions. The analytes were normalized by their isotopically labelled internal standards and quantified with their corresponding calibration curve.

#### **Clinical study: Plasma hydro-soluble vitamins analysis by LC-MSMS**

Sample preparation, including calibration curves, quality controls (QC) and study samples, was automatically performed on a Microlab Star M liquid handler (Hamilton, Reno, NV, USA). Briefly, samples were thawed at room temperature, vortexed, transferred to polypropylene plates containing internal standards, ascorbic Acid (AsC), and DL-dithiothreitol (DTT). Protein was precipitated with 7.5% trichloroacetic acid (TCA). After centrifugation at 2500 rpm for 10 min, the supernatant was filtered through an AcroPrep Advance 96 filter plate (0.2 µm) (Pall)

The analyses were performed on an Acquity *I*-class UPLC system (Waters) hyphenated to a Xevo TQ-XS triple quadrupole mass spectrometer (Waters). Separations were performed on an ACE Excel C_18_-PFP column (100 x 2.1 mm, 2μm, ACE) with a gradient using 5% acetic acid with 0.2% heptafluorobutyric acid (HFBA) in Milli-Q water (Merck®), and acetonitrile as mobile phases. Data was acquired using MassLynx software (Waters), and chromatographic peaks were integrated with TargetLynx (Waters).

#### **Clinical study: Serum amino acid analysis by LC-MSMS**

All sample preparations, involving a derivatization step, were automated and carried out on a Microlab Star M liquid handler (Hamilton). Briefly, plasma samples were thawed at room temperature, vortexed, transferred to a polypropylene plate, and precipitated with a solution containing labeled internal standards in methanol + 0.1% formic acid (FA). After centrifugation (2500 rpm, 10 min), the supernatant was collected for derivatization in borate buffer (pH 8.8) with Aminoquinolyl-*N*-hydroxysuccinimidyl carbamate at 55°C for 10 min under agitation at 500 rpm. Finally, samples were diluted 50 times with a 10 mM ammonium formate and 0.1% FA prior to LC-MSMS analysis. Amino acid analyses were performed on an Acquity *I*-class UPLC system (Waters) hyphenated to a Xevo TQ-XS triple quadrupole mass spectrometer (Waters). Separations were performed on an AccQtag Ultra C_18_ column (2.1 x 100 mm, 1.7 μm, Waters Milford, MA, USA). Data was analyzed with MassLynx software (Waters), and chromatographic peaks were integrated with TargetLynx (Waters).

#### **Clinical study: Clinical chemistry analysis in serum**

Serum samples were directly analyzed without prior preparation. Analyses were carried out on an Architect Ci4100 analyzer (Abbott) composed of a C4000 clinical chemistry module and an i1000SR immunoassay module. Specific kits validated on human serum were used and processed according to the manufacturer. Samples exceeding the upper limit of quantification were diluted by a factor 2 according to the recommended instructions and reanalyzed.

#### **Clinical study: Untargeted plasma metabolomics analysis**

Plasma samples were thawed and 15 µL were transferred to Eppendorf tubes. Proteins were precipitated with 500 µL cold -20°C acetonitrile/methanol/H_2_O (40/40/20, v/v/v) containing isotopically labelled standards, vortexed, and centrifuged at 4°C for 10 minutes at 15’000 rpm. 450 µL of the supernatants were dried in a vacuum centrifuge at 4°C and 5 mbar. Quality control samples were generated by pooling 15 µL of each plasma sample. Volumes of 5, 10, 15, 20 and 25 µL of the pool were taken and extracted as described above. The dried samples were resuspended with 35 µL of acetonitrile/ H_2_O (70/30, v/v) prior to analysis by LC-MS. The LC system (Vanquish UHPLC, Thermo Scientific) was connected to an orbitrap mass spectrometer (Orbitrap Fusion Lumos Tribrid, Thermo Scientific) equipped with a heated electrospray ionisation (H-ESI) source operating in negative and positive ionization mode. Three microliters of each sample were injected into a hydrophilic interaction chromatography (HILIC) analytical column (2.1 mm x 100 mm, 5 µm pore size, 200Å ZIC-pHILIC), guarded by a pre-column (2.1 mm x 20 mm, 200Å ZIC-pHILIC Guard Kit) operating at 35 °C. The software Xcalibur v4.1.31.9 (Thermo Scientific) was used for instrument control and data acquisition. For processing, the Thermo RAW files were converted into centroided mzXML files by using MSConvert (version 3.0.20037) from ProteoWizard (2). Further, an internal automated pipeline consisting of a wrap-up of R (version 3.6.2) (3) packages was used for the pre-processing, peak-picking, feature reduction and putative annotation of the untargeted metabolomics dataset as described below. As a result, 847 metabolomic features were reported for downstream analysis. The untargeted plasma dataset was log2 transformed to achieve a normal distribution for further analysis.

#### **Clinical study: Untargeted plasma metabolomics data analysis**

The data processing was performed on Thermo RAW files, which were converted into centroided mzXML files with MSConvert (version 3.0.20037) from ProteoWizard (2) For the pre-processing, peak-picking, and feature reduction of the metabolomics data an internal automated pipeline consisting of a wrap-up of R (version 3.6.2) (3) packages was applied. This included the XCMS (4) and CAMERA R packages (5) for peak picking, grouping and alignment. A final output consisting of a matrix table with the peak areas for each feature per sample was obtained. The features were further normalized to isotopically labelled internal standards, which were selected based on signal linearity of the pooled quality control samples according to sample amount (6). Specifically, each metabolic feature of four amounts of pooled plasma was normalized to each internal standard and a correlation analysis was performed. The internal standard that resulted in the best correlation between normalized feature and plasma amount was selected for later normalization. Features, where normalization did not result in a linear response after the correlation analysis (r2 < 0.8) were excluded from further data analysis. In addition, features with a coefficient of variation of more than 15 % in the quality control samples were excluded. Finally, 847 metabolic features were kept for further analysis and putatively annotated by matching their exact mass to the Human Metabolome (HDMB) database (https://hmdb.ca/).

#### **Ex vivo experiment: Substrates and pre-digestion**

Briefly, test products (or distilled H2O for NSC and GOS) were subjected to oral, gastric and small intestinal digestion according to the INFOGEST 2.0 method (7). The protocol was modified by implementing six enzyme assays (amylase, pepsin activity, lipase, trypsin, chymotrypsin) together with an assay to quantify bile acids. The protocol additionally implemented the removal of oxygen along the small intestinal incubation and a simulation of the small intestinal absorption via the use of dialysis membranes digestion method to make it compatible with colonic fermentation experiments.

The milk matrices were dosed in the oral phase. To ensure compatibility with colonic incubations, modifications were implemented such as simulation of small intestinal absorption. There was a necessity to remove lactose and digestible proteins from the milk matrices. In contrast, due to its small size, GOS was also partially absorbed along the simulated upper GIT (i.e. for 66.2 %). This erroneously removed fraction along the upper GIT (GOS is known to be indigestible and reach the colon) was re-dosed at the start of the colonic incubation to test 100% of the GOS that was present in the original milk matrix. An equivalent amount of GOS as present in N milk was dosed in the GOS study arm (4.31 g/L).

#### **Ex vivo experiment: Colonic incubations**

#### At the start of the colonic incubations, individual fecal samples were processed in a bioreactor management device (Cryptobiotix, Ghent, Belgium). Each bioreactor contained 5 mL of a blend of small intestine-derived suspension, nutritional medium (M0017, Cryptobiotix, Ghent, Belgium) and a fecal inoculum from a single donor. Bioreactors were sealed individually and rendered anaerobic. After preparation, bioreactors were incubated under continuous agitation (140 rpm) at 37°C (MaxQ 6000, Thermo Scientific, Merelbeke, Belgium).

#### Both blank and probiotic-treated bioreactors were tested across 12 different subjects, with multiple technical replicates harvested either at 0 h (blank only) or 24 h. Gas pressure measurements were taken, and liquid samples were collected for subsequent analysis.

#### **References**

1. Valdivia-Garcia MA, Chappell KE, Camuzeaux S, Olmo-Garcia L, van der Sluis VH, Radhakrishnan ST, et al. Improved quantitation of short-chain carboxylic acids in human biofluids using 3-nitrophenylhydrazine derivatization and liquid chromatography with tandem mass spectrometry (LC-MS/MS). J Pharm Biomed Anal. 2022;221:115060. Epub 20220915. doi: 10.1016/j.jpba.2022.115060. PubMed PMID: 36166933.

2. Chambers MC, Maclean B, Burke R, Amodei D, Ruderman DL, Neumann S, et al. A cross-platform toolkit for mass spectrometry and proteomics. Nat Biotechnol. 2012;30(10):918-20. doi: 10.1038/nbt.2377. PubMed PMID: 23051804; PubMed Central PMCID: PMC3471674.

3. Team RC. R: A Language and Environment for Statistical Computing. Vienna, Austria: R Foundation for Statistical Computing; 2024.

4. Smith CA, Want EJ, O'Maille G, Abagyan R, Siuzdak G. XCMS: processing mass spectrometry data for metabolite profiling using nonlinear peak alignment, matching, and identification. Anal Chem. 2006;78(3):779-87. doi: 10.1021/ac051437y. PubMed PMID: 16448051.

5. Kuhl C, Tautenhahn R, Bottcher C, Larson TR, Neumann S. CAMERA: an integrated strategy for compound spectra extraction and annotation of liquid chromatography/mass spectrometry data sets. Anal Chem. 2012;84(1):283-9. Epub 20111212. doi: 10.1021/ac202450g. PubMed PMID: 22111785; PubMed Central PMCID: PMC3658281.

6. Boysen AK, Heal KR, Carlson LT, Ingalls AE. Best-Matched Internal Standard Normalization in Liquid Chromatography-Mass Spectrometry Metabolomics Applied to Environmental Samples. Anal Chem. 2018;90(2):1363-9. Epub 20180103. doi: 10.1021/acs.analchem.7b04400. PubMed PMID: 29239170.

7. Brodkorb A, Egger L, Alminger M, Alvito P, Assuncao R, Ballance S, et al. INFOGEST static in vitro simulation of gastrointestinal food digestion. Nat Protoc. 2019;14(4):991-1014. Epub 20190318. doi: 10.1038/s41596-018-0119-1. PubMed PMID: 30886367.
