# Supplementary material for: The Impact of Low-Lactose, High Galacto-Oligosaccharides Milk on Gut Microbiome and Plasma Metabolome in Healthy Adults: A Randomized, Double-Blind, Controlled Clinical Trial Complemented by Ex Vivo Experiments
Source: Curr Dev Nutr. 2025 Jul 24;9(9):107506. doi: 10.1016/j.cdnut.2025.107506 (PMC12405628; doi:10.1016/j.cdnut.2025.107506)
Supplement: Multimedia component 2 [file mmc2.docx]

The impact of low-lactose, high galacto-oligosaccharides (GOS) milk on gut microbiome and plasma metabolome in healthy adults: A randomized, double-blind, controlled clinical trial complemented by ex vivo experiments.

Siegwald et al.

**Supplementary Figure 1. Diagram of study design and study visit procedures**

FFQ, Food Frequency Questionnaire; GSRS, Gastrointestinal Symptom Rating Scale; SF36, Short-Form 36; STAI, State-Trait Anxiety Inventory; STAI-T, situational anxiety, STAI-S: state anxiety; V, visit.

The impact of low-lactose, high galacto-oligosaccharides (GOS) milk on gut microbiome and plasma metabolome in healthy adults: A randomized, double-blind, controlled clinical trial complemented by ex vivo experiments.

Siegwald et al.


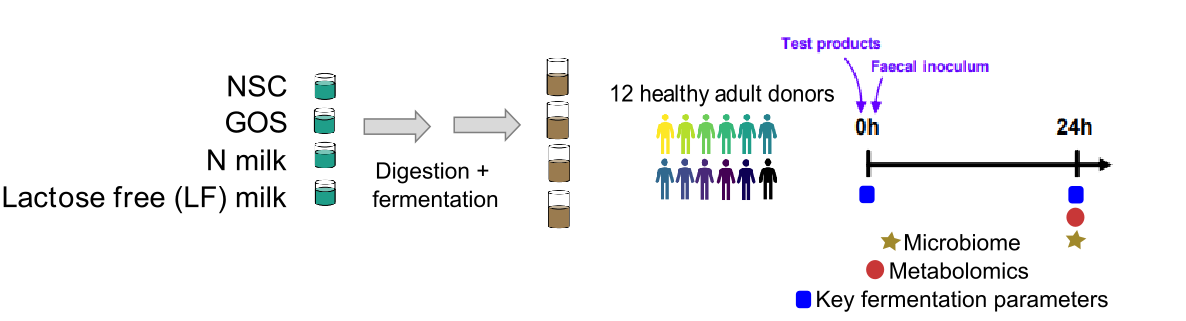


**Supplementary Figure 2. Ex-vivo batch fermentation experimental design.**

The tested conditions included no substrate control (NSC), lactose free milk, GOS, and N milk. Conditions containing milk products underwent a digestion and fermentation step. The dose of GOS alone and GOS within N milk was 4.3g/L. No residual lactose was detected in N milk after the digestion step. Fecal samples from 12 adult healthy donors were introduced into individual vessels for batch fermentation experiments. Media samples were collected at 0h for NSC and at 24h of fermentation for all conditions. key fermentation analyses were measured at 0h for NSC while microbiome, metabolomic analyses were performed at 24h for all conditions.

The impact of low-lactose, high galacto-oligosaccharides (GOS) milk on gut microbiome and plasma metabolome in healthy adults: A randomized, double-blind, controlled clinical trial complemented by ex vivo experiments.

Siegwald et al.

(n=12)

(n=1 dropped out due to abdominal cramps)

## Allocated to investigation

## Allocated to control

(n=11)

(n=12)

## Allocated to control

## Allocated to investigation

(n=12)

Analysed (n=23)

## Analysis

Excluded (n=9)

♦  Not meeting inclusion criteria (n=7)

♦  Declined to participate (n=1)

♦  Sickness (n=1)

## Enrollment

Assessed for eligibility (n=33)

Randomized (n=24)

**Supplementary Figure 3. CONSORT 2010 Flow.**

The impact of low-lactose, high galacto-oligosaccharides (GOS) milk on gut microbiome and plasma metabolome in healthy adults: A randomized, double-blind, controlled clinical trial complemented by ex vivo experiments.

Siegwald et al.


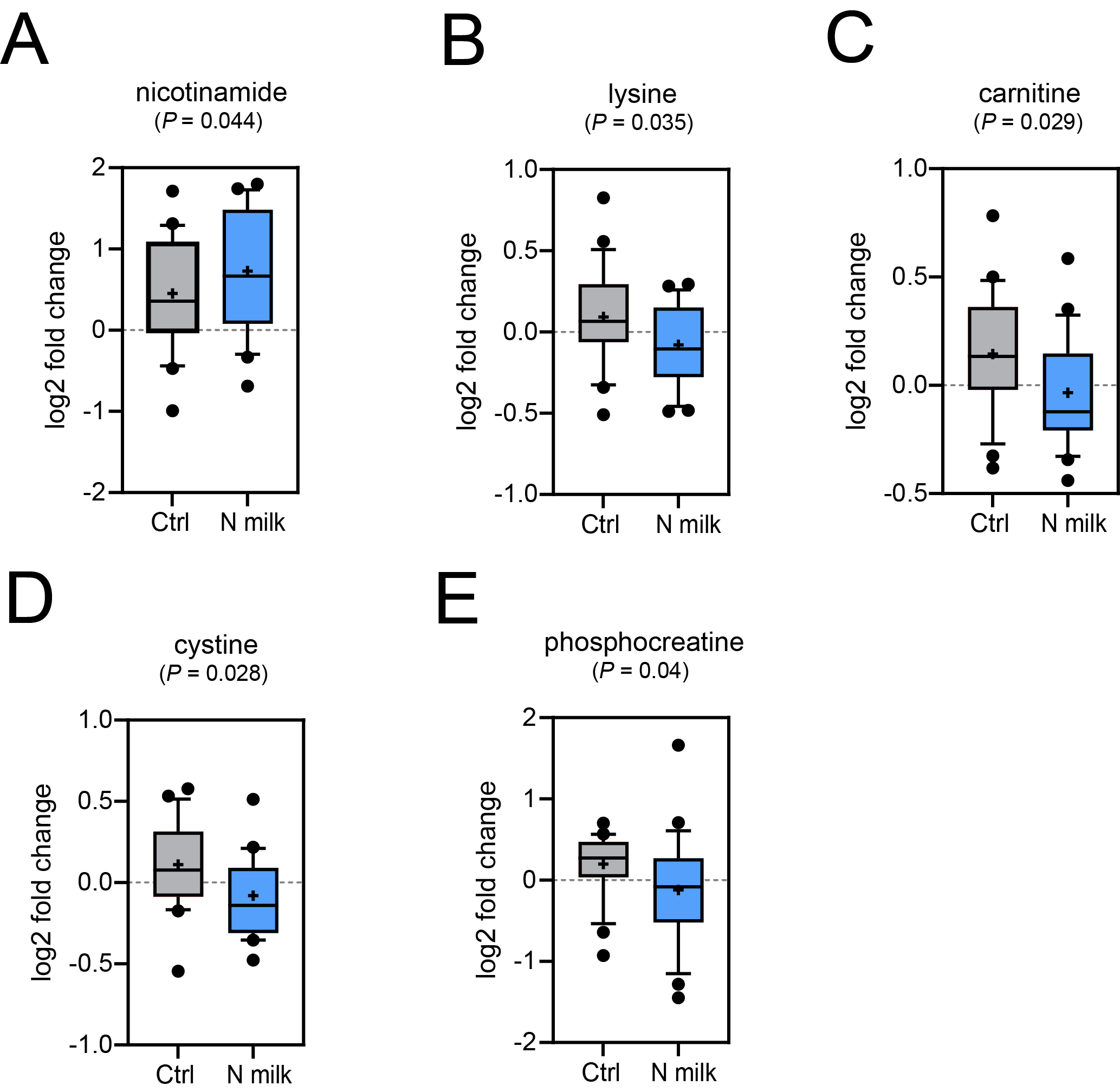


**Supplementary Figure 4**. **Impact of the intervention on selected metabolites**.

The impact of low-lactose, high galacto-oligosaccharides (GOS) milk on gut microbiome and plasma metabolome in healthy adults: A randomized, double-blind, controlled clinical trial complemented by ex vivo experiments.

Siegwald et al.


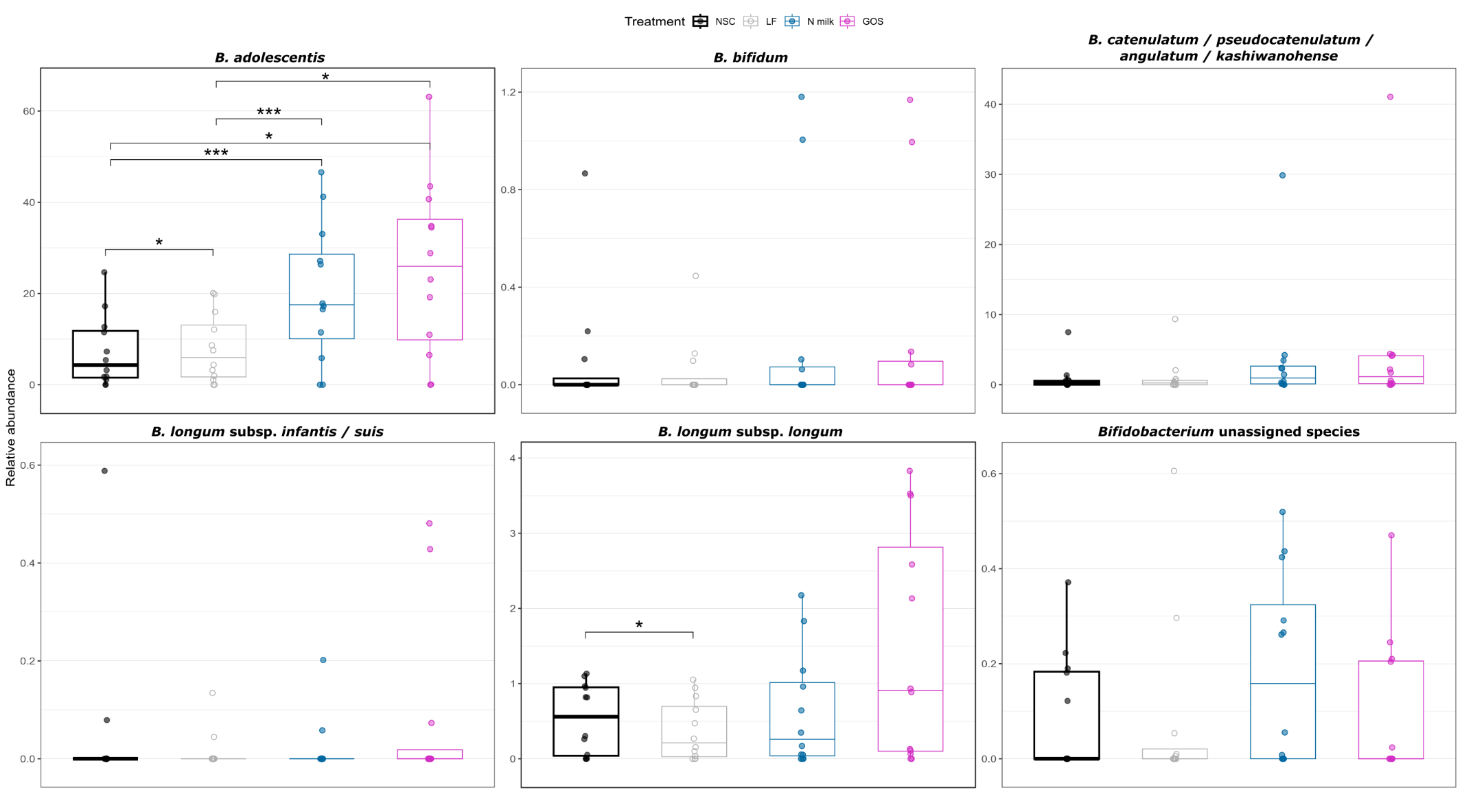


**Supplementary Figure 5**. In vitro fermentation: Impact of treatment on individual *Bifidobacterium* species / subspecies. Statistical differences between groups are visualized via * (0.01 < FDR-adjusted *P* value < 0.05), ** (0.001 < FDR-adjusted *P* value < 0.01) or *** (FDR-adjusted *P* value < 0.001).
