# Supplementary material for: The Impact of Low-Lactose, High Galacto-Oligosaccharides Milk on Gut Microbiome and Plasma Metabolome in Healthy Adults: A Randomized, Double-Blind, Controlled Clinical Trial Complemented by Ex Vivo Experiments
Source: Curr Dev Nutr. 2025 Jul 24;9(9):107506. doi: 10.1016/j.cdnut.2025.107506 (PMC12405628; doi:10.1016/j.cdnut.2025.107506)
Supplement: Multimedia component 3 [file mmc3.docx]

The impact of low-lactose, high galacto-oligosaccharides (GOS) milk on gut microbiome and plasma metabolome in healthy adults: A randomized, double-blind, controlled clinical trial complemented by ex vivo experiments.

Siegwald et al.

| **Supplementary Table 1. Plasma compounds analyzed with targeted methods** |
| --- |
| - Short chain fatty acids, medium chain fatty acids, and organic acids: lactate, acetate, propionate, butyrate, valerate, isobutyrate, isovalerate, 3-methylvalerate, hexanoic acid, octanoic acid, decanoic acid, 3-hydroxybutyrate, and acetoacetate - Water-soluble vitamins: riboflavin, pantothenic acid, pyridoxal, pyridoxic acid, folic acid, 5-methyltetrahydrofolate (5-mTHF), pyridoxine, pyridoxamine, pyridoxal-5 phosphate (PLP), thiamine, nicotinamide, nicotinuric acid, nicotinic acid and 4-aminobenzoyl glutamic acid (4-ABGA). - Amino acids: alanine, leucine, isoleucine, beta-alanine, lysine, sarcosine, methionine, arginine, ornithine, phenylalanine, asymmetric dimethylarginine (ADMA), aymmetric dimethylarginine (SDMA), proline, serine, asparagine, taurine, aspartic acid, threonine, citrulline, tryptophan, glutamic acid, tyrosine, glutamine, valine, glycine, trans-4-hydroxyproline (hydroxyproline), histidine, 1-methylhistidine, alpha-aminobutyric acid (AABA), and 3-methyl-L-histidine. - Additional analytes: holotranscobalamin, folate, homocysteine, magnesium, and copper. |

| **Supplementary Table 2. Baseline demographic characteristics of participants (N=24)** | |
| --- | --- |
|  | Mean (standard deviation) |
| **Female:Male** | 17:7 |
| **Age (years)** | 35.8 (7.02) |
| **Body mass index (BMI) (kg/m2)** | 22.6 (2.56) |
| **Weight (kg)** | 63.9 (11.66) |

The impact of low-lactose, high galacto-oligosaccharides (GOS) milk on gut microbiome and plasma metabolome in healthy adults: A randomized, double-blind, controlled clinical trial complemented by ex vivo experiments.

Siegwald et al.The impact of low-lactose, high galacto-oligosaccharides (GOS) milk on gut microbiome and plasma metabolome in healthy adults: A randomized, double-blind, controlled clinical trial complemented by ex vivo experiments.

Siegwald et al.

| **Supplementary Table 3. Baseline dietary intake of participants (N=24)** | |
| --- | --- |
|  | **Mean (Standard Deviation)** |
| **Energy (kcal)** | 1380.91 (543.62) |
| **Carbohydrate (kcal)** | 576.14 (253.31) |
| **Carbohydrate (%)** | 41.90 (6.75) |
| **Fats (kcal)** | 531.93 (219.17) |
| **Fats (%)** | 38.52 (4.81) |
| **Saturated fats (kcal)** | 187.393 (87.16) |
| **Saturated fats (%)** | 13.42 (2.48) |
| **Protein (kcal)** | 229.79 (102.17) |
| **Protein (%)** | 16.72 (3.81) |
| **Dietary fibre (g)** | 12.06 (5.87) |
| **Dietary fibre (%)** | 0.90 (0.35) |
| %, percentage of total energy intake. Data based on food frequency questionnaires | |

The impact of low-lactose, high galacto-oligosaccharides (GOS) milk on gut microbiome and plasma metabolome in healthy adults: A randomized, double-blind, controlled clinical trial complemented by ex vivo experiments.

Siegwald et al.

**Supplementary Table 4.** Clinical study – Microbiome richness, diversity, taxonomic and functional compositions.

Accessible on Figshare under the DOI: 10.6084/m9.figshare.28807100
