# Supplementary material for: The Impact of Low-Lactose, High Galacto-Oligosaccharides Milk on Gut Microbiome and Plasma Metabolome in Healthy Adults: A Randomized, Double-Blind, Controlled Clinical Trial Complemented by Ex Vivo Experiments
Source: Curr Dev Nutr. 2025 Jul 24;9(9):107506. doi: 10.1016/j.cdnut.2025.107506 (PMC12405628; doi:10.1016/j.cdnut.2025.107506)
Supplement: Multimedia component 5 [file mmc5.docx]

The impact of low-lactose, high galacto-oligosaccharides (GOS) milk on gut microbiome and plasma metabolome in healthy adults: A randomized, double-blind, controlled clinical trial complemented by ex vivo experiments.

Siegwald et al.

| **Supplementary Table 6. Short chain fatty acids concentration in feces** | | | | | | | |
| --- | --- | --- | --- | --- | --- | --- | --- |
|  | **Control (n=24)** | | **Intervention (n=23)** | |  | | |
|  | Baseline | Post-treatment | Baseline | Post-treatment | *P* value | 95% CI | Cohen’s d |
| Acetate (μM/g) | 303.6 (166.4) | 409.2 (434.9) | 464.8 (400.5) | 348.7 (280.9) | 0.6757 | -257.04; 170.3 | 0.0975 |
| Butyrate (μM/g) | 61.3 (33.0) | 71.1 (57.7) | 73.0 (54.5) | 60.6 (37.6) | 0.4824 | -41.54; 20.35 | 0.1644 |
| Propionate (μM/g) | 102.4 (53.5) | 113.7 (101.5) | 111.6 (79.3) | 106.7 (80.7) | 0.8477 | -64.05; 53.18 | 0.0459 |
| Valerate (μM/g) | 10.0 (4.6) | 12.0 (8.2) | 13.7 (11.4) | 10.2 (5.2) | 0.4550 | -6.4; 2.99 | 0.1800 |

Values are expressed as means and standard deviations. 95% Confidence Intervals and *P* value refer to the difference between the change from baseline in both arms.

The impact of low-lactose, high galacto-oligosaccharides (GOS) milk on gut microbiome and plasma metabolome in healthy adults: A randomized, double-blind, controlled clinical trial complemented by ex vivo experiments.

Siegwald et al.

| **Supplementary Table 7. Short chain fatty acid and other analytes concentrations in plasma** | | | | | | | |
| --- | --- | --- | --- | --- | --- | --- | --- |
|  | **Control (n=24)** | | **N milk (n=23)** | |  | | |
|  | Baseline | Post-treatment | Baseline | Post-treatment | *P* value | 95% CI | Cohen’s d |
| Acetate (μM) | 102.0 (182.71) | 66.0 (28.39) | 61.9 (23.33) | 80.9 (40.84) | 0.0211 | 3.28; 35.95 | 0.5768 |
| Butyrate (μM) | 0.9 (0.42) | 0.8 (0.40) | 0.8 (0.32) | 0.8 (0.38) | 0.3467 | -0.076;0.207 | 0.2214 |
| Propionate (μM) | 1.2 (0.28) | 1.3 (0.39) | 1.3 (0.43) | 1.3 (0.39) | 0.4365 | -0.123;0.273 | 0.1824 |
| Valerate (μM) | 0.1 (0.04) | 0.1(0.04) | 0.2 (0.06) | 0.1 (0.05) | 0.8639 | -0.026;0.0302 | 0.0399 |
| Lactate (μM) | 1033 (487.60) | 898.4 (372.35) | 1052.8 (354) | 934.9 (325.82) | 0.7239 | -129.54;183.1 | 0.0823 |
| Total SCFA (μM) | 65.52 (19.92) | 68.16 (28.82) | 64.20 (23.58) | 83.22 (41.17) | 0.0210 | 3.33; 36.28 | 0.5772 |
| Acetoacetate (pM) | 34.96 (19.658) | 30.74 (14.226) | 31.76 (20.320) | 44.96 (41.778) | 0.0866 | -2.218;30.257 | 0.4146 |
| Holotranscobalamin (pM) | 74.63 (23.550) | 86.11 (30.560) | 74.92 (30.141) | 76.60 (26.757) | 0.0431 | -18.44;-0.325 | 0.4974 |
| Nicotinamide (μg/L) | 32.36 (12.208) | 52.43 (34.431) | 30.03 (9.735) | 70.30 (43.276) | 0.0245 | 2.188;28.394 | 0.5603 |
| Beta-alanine (μM) | 3.37 (0.680) | 3.15 (0.533) | 3.33 (0.681) | 3.66 (0.794) | 0.0077 | 0.162;1.912 | 0.7324 |
| Octanoic acid (μM) | 0.93 (0.462) | 0.74 (0.223) | 0.79 (0.298) | 0.85 (0.306) | 0.0252 | 0.0207;0.278 | 0.5575 |

Values are expressed as means and standard deviations. 95% Confidence Intervals and *P* value refer to the difference between the change from baseline in both arms.

The impact of low-lactose, high galacto-oligosaccharides (GOS) milk on gut microbiome and plasma metabolome in healthy adults: A randomized, double-blind, controlled clinical trial complemented by ex vivo experiments.

Siegwald et al.

| **Supplementary Table 8. Questionnaire-based endpoints** | | | | | | | |  |
| --- | --- | --- | --- | --- | --- | --- | --- | --- |
|  | **Control (n=23)** | | **N milk (n=23)** | |  | | |  |
|  | Baseline | Post-treatment | Baseline | Post-treatment | *P* value | 95% CI | Cohen’s d |  |
| Bristol stool scale | 3.73 (1.077) | 3.59 (1.008) | 3.35 (1.112) | 3.48 (1.201) | 0.9712 | -0.59; 0.61 | 0.00840 | |
| STAI | 33.57 (7.662) | 34.43 (8.862) | 32.83 (7.791) | 32.43 (8.005) | 0.4875 | -5.4; 2.66 | 0.1582 | |
| SF36-Energy /Fatigue Score | 67.39 (13.044) | 68.70 (12.175) | 69.78 (12.105) | 68.70 (13.586) | 0.3473 | -5.28; 1.95 | 0.2152 | |
| SF36-Emotional Problems Score | 89.86 (21.165) | 76.81 (39.485) | 86.96 (31.365) | 88.41 (21.576) | 0.0851 | -2.01; 28.52 | 0.4050 | |
| SF36-Emotional Well-being Score | 76.52 (10.672) | 78.96 (14.230) | 78.43 (12.225) | 76.87 (16.089) | 0.0512 | -8.23; 0.02 | 0.4638 | |
| SF36-General Health Score | 76.30 (11.795) | 76.52 (12.380) | 75.65 (12.089) | 74.13 (12.215) | 0.2406 | -4.9; 1.3 | 0.2705 | |
| SF36-Pain Score | 95.11 (6.238) | 87.83 (18.743) | 93.15 (12.322) | 92.28 (10.333) | 0.1078 | -1.41; 13.26 | 0.3765 | |
| SF36-Physical Functioning Score | 98.70 (2.704) | 97.83 (6.541) | 99.13 (4.170) | 99.57 (2.085) | 0.2683 | -1.3; 4.44 | 0.2546 | |
| SF36-Physical Health Score | 95.65 (9.689) | 93.48 (21.608) | 94.57 (14.993) | 91.30 (16.182) | 0.7605 | -15.05; 11.17 | 0.0691 | |
| SF36-Social Functioning Score | 95.65 (8.926) | 90.22 (17.253) | 89.67 (12.865) | 89.67 (13.926) | 0.7334 | -4.69; 6.55 | 0.0772 | |
| GSRS | 1.48 (0.390) | 1.41 (0.334) | 1.38 (0.356) | 1.55 (0.377) | 0.0048 | 0.06; 0.31 | 0.7093 | |

Values are expressed as means and standard deviations. 95% Confidence Intervals and *P* value refer to the difference between the change from baseline in both arms.
